# Regulation of mRNA Abundance by Polypyrimidine Tract-Binding Protein-Controlled Alternate 5′ Splice Site Choice

**DOI:** 10.1371/journal.pgen.1004771

**Published:** 2014-11-06

**Authors:** Fursham M. Hamid, Eugene V. Makeyev

**Affiliations:** 1 School of Biological Sciences, Nanyang Technological University, Singapore; 2 MRC Centre for Developmental Neurobiology, King's College London, London, United Kingdom; International Centre for Genetic Engineering and Biotechnology, Italy

## Abstract

Alternative splicing (AS) provides a potent mechanism for increasing protein diversity and modulating gene expression levels. How alternate splice sites are selected by the splicing machinery and how AS is integrated into gene regulation networks remain important questions of eukaryotic biology. Here we report that polypyrimidine tract-binding protein 1 (Ptbp1/PTB/hnRNP-I) controls alternate 5′ and 3′ splice site (5′ss and 3′ss) usage in a large set of mammalian transcripts. A top scoring event identified by our analysis was the choice between competing upstream and downstream 5′ss (u5′ss and d5′ss) in the exon 18 of the *Hps1* gene. *Hps1* is essential for proper biogenesis of lysosome-related organelles and loss of its function leads to a disease called type 1 Hermansky-Pudlak Syndrome (HPS). We show that Ptbp1 promotes preferential utilization of the u5′ss giving rise to stable mRNAs encoding a full-length Hps1 protein, whereas bias towards d5′ss triggered by Ptbp1 down-regulation generates transcripts susceptible to nonsense-mediated decay (NMD). We further demonstrate that Ptbp1 binds to pyrimidine-rich sequences between the u5′ss and d5′ss and activates the former site rather than repressing the latter. Consistent with this mechanism, u5′ss is intrinsically weaker than d5′ss, with a similar tendency observed for other genes with Ptbp1-induced u5′ss bias. Interestingly, the brain-enriched Ptbp1 paralog Ptbp2/nPTB/brPTB stimulated the u5′ss utilization but with a considerably lower efficiency than Ptbp1. This may account for the tight correlation between Hps1 with Ptbp1 expression levels observed across mammalian tissues. More generally, these data expand our understanding of AS regulation and uncover a post-transcriptional strategy ensuring co-expression of a subordinate gene with its master regulator through an AS-NMD tracking mechanism.

## Author Summary

Mammalian gene expression is extensively controlled at the post-transcriptional level and understanding of the underlying mechanisms can provide important biomedical insights. Here we identified a number of novel alternate splicing (AS) events where the choice between competing splice sites (ss) is regulated by polypyrimidine tract-binding protein 1 (Ptbp1/PTB/hnRNP-I). A top-scoring event was the choice between alternate upstream and downstream 5′ss (u5′ss and d5′ss) in the *Hps1* gene mutated in patients with type 1 Hermansky-Pudlak Syndrome (HPS). Preferential utilization of the u5′ss in the presence of Ptbp1 gives rise to stable mRNAs encoding a full-length Hps1 protein, whereas the d5′ss bias triggered by Ptbp1 down-regulation generates RNA species cleared by nonsense-mediated decay (NMD). We show that Ptbp1 functions in this circuitry by activating the intrinsically weaker u5′ss. Brain-enriched Ptbp1 paralog Ptbp2/nPTB/brPTB stimulated the u5′ss utilization but with a considerably lower efficiency than Ptbp1. We propose that this mechanism accounts for a tight correlation between Hps1 with Ptbp1 expression levels observed in mammalian tissues. Overall, these data expand our understanding of AS regulation and uncover an AS-NMD-mediated tracking mechanism ensuring co-expression of master regulator and its subordinate gene.

## Introduction

Eukaryotes rely on post-transcriptional control of their gene expression programs to a remarkable extent. A compelling example of this trend is the ability of many mammalian transcripts to undergo alternative splicing (AS) regulated by interplay between RNA-encoded *cis*-elements and *trans*-acting factors [1]–[4]. Distinct AS patterns include singular and mutually exclusive cassette exons, alternative 5′- and 3′-terminal exons, intron retention events, and alternate 5′ and 3′ splice site (5′ss and 3′ss) choice [1], [5]. Of these, the latter two categories (A5C and A3C), involve alternative utilization of exonic termini and constitute a major part of tissue-specific AS programs [6], [7]. Many of these events are known to have biologically and medically important functions (e.g., [8], [9] and references therein).

Several earlier studies have begun elucidating molecular mechanisms involved in A5C regulation. Important factors affecting recognition of 5′ss include (1) intrinsic efficiencies, or strengths with which these elements interact with the U1 snRNP component of the spliceosome and (2) the presence of adjacent splicing silencer or enhancer sequences that can modulate AS outcomes by recruiting cognate *trans*-regulators [10]. A common A5C regulation strategy relies on a splicing silencer positioned between an upstream and a downstream 5′ss alternatives (u5′ss and d5′ss) [11], [12]. This often stimulates the u5′ss though silencer-dependent repression of the competing d5′ss [11], [12]. However, the situation is complicated by the fact that hnRNP family proteins interacting with classical splicing silencers may additionally activate splicing reaction when recruited downstream of a 5′ss [13]. Thus, hnRNP binding between u5′ss and d5′ss alternatives could theoretically bias A5C towards the u5′ss by either repressing the d5′ss, stimulating the u5′ss or both. It is generally unclear which of these three possibilities is realized in natural contexts since most published studies on A5C regulation mechanisms rely largely on recombinant or/and *in vitro* approaches [11]–[13].

Mounting evidence suggests that in addition to generating multiple protein isoforms from a single gene [2], [14] AS is widely used to control gene expression levels [4], [15], [16]. A prevalent post-transcriptional mechanism regulating mRNA abundance involves coupling between AS and nonsense-mediated decay (NMD), a quality control mechanism targeting mRNAs containing premature translation termination codons (PTCs) for degradation [17], [18]. AS-NMD plays important roles in diverse biological processes [19] including regulation of RNA-binding protein expression [20], [21], granulocyte development [22], axonal guidance [23] and brain response to seizures [24].

An hnRNP family member called polypyrimidine tract-binding protein 1 (Ptbp1/PTB/hnRNP I; [25], [26]) is known to control expression of several genes through AS-NMD. Ptbp1 homeostasis in proliferating cells is maintained by an AS-NMD-mediated auto-regulation mechanism [27]. Dampening Ptbp1 levels in neurons by microRNA miR-124 triggers global changes in cellular AS patterns and leads to increased expression of at least three AS-NMD targets encoding brain-enriched Ptbp1 paralog Ptbp2/nPTB/brPTB and post-synaptic proteins Gabbr1 and PSD-95/Dlg4 [28]–[30]. These genes contain Ptbp1-repressible cassette exons essential for open reading frame (ORF) integrity. Skipping of these exons in the presence of Ptbp1 results in a frame-shift and triggers NMD. On the other hand, their inclusion upon Ptbp1 down-regulation leads to accumulation of translationally active mRNAs. It is currently unknown whether the repertoire of Ptbp1-dependent AS-NMD targets is limited to brain-enriched mRNAs or if it could additionally include other types of transcripts, e.g. those undergoing down-regulation during nervous system development.

Here we carried out a systematic analysis of transcriptome-wide RNA sequencing (RNA-seq) data and uncovered a large repertoire of Ptbp1-regulated A5C and A3C targets. Strikingly, one of the newly identified A5C events participates in an unusual AS-NMD circuitry controlling the abundance of mRNA encoding Hps1, a subunit of the Rab32/38 guanine nucleotide exchange factor (GEF) essential for biogenesis of lysosome-related organelles and mutated in patients with Hermansky-Pudlak Syndrome (HPS; OMIM: 203300; [31]–[34]). We describe the mechanistic underpinning of this regulation and provide evidence that this post-transcriptional mechanism may play an important part in shaping Hps1 tissue-specific expression patterns.

## Results

### Ptbp1 controls a number of alternate 5′ and 3′ splice site events

**Figure 1:**
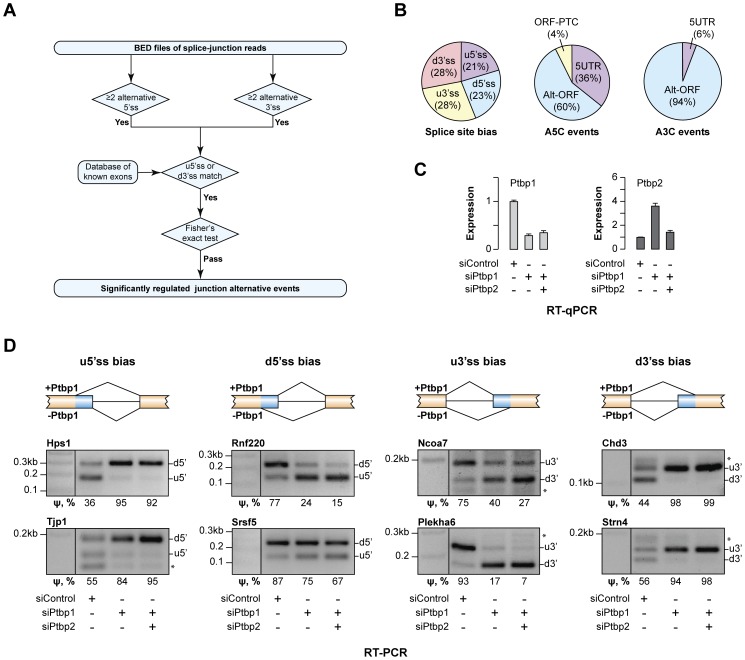
Ptbp1 regulates the choice between alternate 5′ and 3′ splice sites for an extensive set of genes. (**A**) Data analysis workflow used to identify regulated A5C and A3C splice junctions. (**B**) Summary of newly identified events regulated by Ptbp1. The pie chart on the left classifies A5C and A3C events based on whether Ptbp1 and Ptbp2 bias the AS choice towards upstream or downstream splice site alternative. The other two pie charts categorize A5C and A3C events according to their effect on mRNA function. (**C**) CAD cells were treated with Ptbp1-specific siRNA (siPtbp1), a mixture of siPtbp1 and Ptbp2-specific siRNA (siPtbp2) or control siRNA (siControl) and the expression levels of Ptbp1 and Ptbp2 mRNAs were analyzed by RT-qPCR. Data are averaged from 3 independent experiments ±SD. (**D**) RT-PCR validation of examples of the four splicing topologies (Ptbp1-biased choice of u5′ss, d5′ss, u3′ss or d3′ss) in CAD samples treated as in (C). ψ (percent spliced in) values shown at the bottom indicate the abundance of the longer splice product isoform as percentage of the total. Data are averaged from 3 independent analyses.

To uncover additional Ptbp1 targets, we adapted a previously described RNA-seq analysis algorithm relying on Fisher's exact test to identify significantly regulated A5C and A3C events (see e.g., [35]; Fig. 1A). After confirming functionality of this approach with training RNA-seq datasets from neuroblastoma CAD and fibrosarcoma L929 cells (Fig. S1 and Tables S1 and S2) we repeated the analysis for our RNA-seq datasets obtained for CAD cells transfected with control siRNA, Ptbp1-specific siRNAs or a mixture of siRNAs against Ptbp1 and its brain-enriched paralog Ptbp2 with a largely overlapping AS regulation preferences [26], [36], [37] (siControl, siPtbp1 and siPtbp1/2, respectively; [29]; NCBI Gene Expression Omnibus accession number GSE37933). This identified 41 A5C and 52 A3C events consistently regulated in both siPtbp1 and siPtbp1/2 samples (Fisher's exact test p<0.05 and >5% difference in the percent spliced in statistic (ψ) [7]; Tables S3 and S4).

The newly identified targets belonged to diverse functional categories and included amongst others regulators of transcription (e.g., *Chd3*, *Ezh2*, *Hmga1*, *Mef2a*, *Msx1*, *Ncoa7* and *Prmt1*) and RNA metabolism (e.g., *Hnrnpc*, *Hnrnph3*, *Larp4*, *Serbp1*, *Son*, *Srsf5* and *Ythdc1*) (Tables S3 and S4). Several target genes such as *Enah*, *Hps1*, *Plekha6*, *Ssna1*, *Strn4* and *Tjp1* were linked to human diseases [32], [33], [38]–[42]. Comparable fractions of the AS events were biased by Ptbp1 towards intron-distal (u5′ss and d3′ss) or intron-proximal sites (d5′ss and u3′ss) and most of these changes either altered ORF without introducing a premature termination codon (PTC) or changed the 5′UTR sequence (Fig. 1B). For two disease-associated genes, *Hps1* and *Ssna1*, A5C was predicted to modulate mRNA abundance through AS-NMD (Fig. 1B).

Of note, Ptbp1 had been previously proposed to regulate the A5C event in the *Usp5* gene identified by our analysis [43]. This positive control and 11 examples of newly identified targets representing the four AS patterns (Ptbp1-induced bias towards u5′ss, d5′ss, u3′ss or d3′ss) were selected for RT-PCR validation. Satisfyingly, all 12 genes showed readily detectable AS changes upon Ptbp1 and Ptbp1/2 knockdown (Fig. 1C–D and Fig. S1B). Ptbp1 knockdown accounted for most of the effect in all targets except Usp5 where Ptbp2 contribution was substantial (Fig. S1B). We concluded that Ptbp1 was involved in large-scale regulation of A5Cs and A3Cs.

### Ptbp1 regulates Hps1 expression through alternate 5′ss choice coupled with NMD

**Figure 2:**
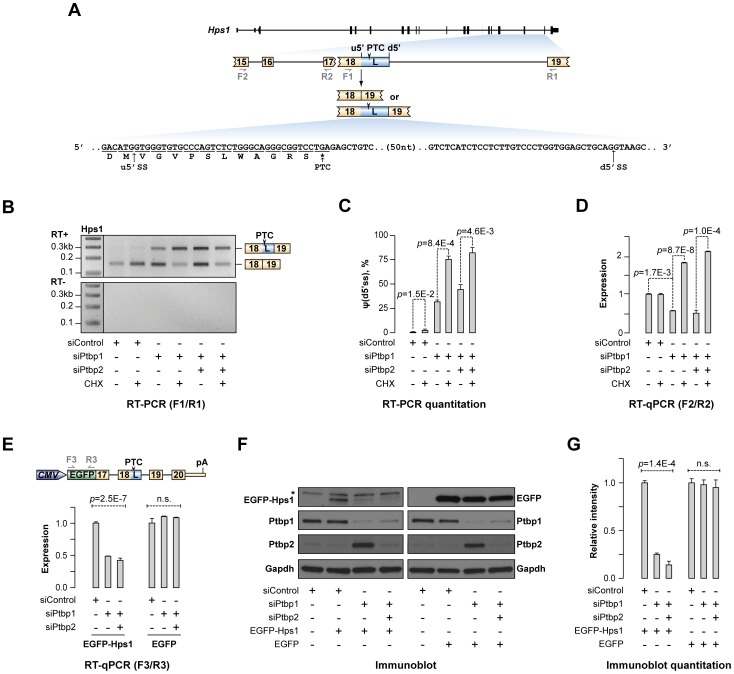
Ptbp1 regulates Hps1 mRNA abundance through AS-NMD. (**A**) *Hps1* gene structure with a close-up of the exon 15-exon 19 segment. The arrowhead indicates premature termination codon (PTC) in the longer (L) isoform of exon 18 and the half-arrows underneath correspond to PCR primers used in this study. PTC-containing sequence between the alternate u5′ss and d5′ss is shown at the bottom. (**B**) CAD cells transfected with siControl, siPtbp1 or siPtbp1/2 were treated with cycloheximide (CHX) or DMSO (control) and the Hps1 splicing pattern was analyzed by RT-PCR with F1/R1 primers. Note that knock-down of Ptbp1 alone or together with Ptbp2 promote utilization of d5′ss and that the corresponding PTC-containing splice product is further stabilized by CHX treatment. (**C**) Relative utilization of the d5′ss quantified from (B). (**D**) RT-qPCR analysis of the CAD samples from (B) with F2/R2 primers shows that reduced expression of Ptbp1 diminishes Hps1 mRNA expression levels and that this effect is rescued by CHX. (**E–G**) 3′-terminal part of the Hps1 gene is sufficient for Ptbp1-dependent control at the protein level. (**E**) *Top*, expression construct encoding EGFP-Hps1 fusion protein. *Bottom*, CAD cells treated with indicated siRNAs were transfected with constructs encoding either EGFP-Hps1 or unmodified EGFP and splicing patterns of the recombinant transcripts were analyzed by RT-qPCR using F3/R3 primers. (**F**) Immunoblot analysis of EGFP-containing proteins in CAD samples prepared as in (E). Ptbp1- and Ptbp2-specific antibodies were used to validate corresponding knockdown efficiencies, whereas Gapdh-specific antibody was used as a lane loading control. (**G**) Quantitation of the results in (**F**). Data in (C, D and G) are averaged from at least three independent experiments ±SD.

Consistently highest Δψ values in the A5C category were detected for the exon 18 of the *Hps1* gene that its homozygous loss-of-function form leads to type 1 HPS (Table S3 and Fig. 1D). Utilization of the u5′ss was expected to generate a full-length Hps1 ORF, whereas splicing at the alternative d5′ss was predicted to generate a PTC-containing version of exon 18 (18L) triggering NMD (Fig. 2A). HPS is currently incurable condition associated with albinism, prolonged bleeding, ceroid storage and frequently, a progressive lung disease limiting patients' lifespan [32]–[34]. Similar symptoms are observed in the *pale ear* mouse model homozygous for a loss-of-function *Hps1* allele [32], [44].

To test if the expression of this medically important gene was indeed controlled by AS-NMD in a Ptbp1-dependent manner, CAD cells pre-treated with siControl, siPtbp1 or siPtbp1/2 were incubated in the presence of either cycloheximide (CHX), an inhibitor of protein synthesis also blocking NMD, or an equal amount of control solution (DMSO) (Fig. 2B–D and S2A). RT-PCR analysis of these samples using F1/R1 primers (Fig. 2B–C) confirmed Ptbp1 dependence of the A5C switch and showed that utilization of the d5′ss was significantly elevated in the presence of CHX (e.g., 2.45-fold increase for siPtbp1-treated samples; p = 8.4×10^−4^), consistent with the sensitivity of the corresponding splice form to NMD. Importantly, RT-quantitative (q) PCR analyses of the above six samples using F2/R2 primers revealed significant down-regulation of the Hps1 mRNA steady-state levels upon Ptbp1 or combined Ptbp1 and Ptbp2 knockdown (Fig. 2D; t-test p = 0.0017 for siControl vs. siPtbp1 and p = 0.025 for siControl vs. siPtbp1/2). This down-regulation effect was completely rescued by CHX treatment (Fig. 2D) indicating that a major fraction of Hps1 transcripts in the siPtbp1 and siPtbp1/2 samples was subjected to NMD. Similar changes in relative abundance of the two A5C forms and Hps1 mRNA expression were detected when we inhibited NMD with siRNA targeting its key component, Upf1 [17], [18] (Fig. S2B–D).

We next wondered if the newly identified post-transcriptional mechanism could modulate Hps1 expression at the protein level. Since immunodetection of the endogenous Hps1 protein is complicated by its relatively low abundance [45], we constructed a CMV promoter-driven plasmid containing an EGFP ORF fused in frame with the relevant 3′-terminal fragment of the *Hps1* gene (Fig. 2E). CAD cells pre-treated with siControl, siPtbp1 or siPtbp1/2 siRNAs were transfected with this construct and analyzed by RT-qPCR and immunoblotting 72 hours post-transfection. RT-qPCR analysis using EGFP-specific primers (F3/R3) showed that, similar to the endogenous Hps1 mRNA, recombinant EGFP-Hps1 transcripts were significantly down-regulated in the siPtbp1 and siPtbp1/siPtbp2 samples (Fig. 2E) and underwent corresponding A5C changes (Fig. S2E). On the other hand, expression levels of a similarly designed control construct containing EGFP ORF but lacking the Hps1 part were virtually unchanged upon Ptbp1 and Ptbp1/2 knockdown (Fig. 2E).

Immunoblotting analysis of the EGFP-Hps1-transfected samples with an EGFP-specific antibody detected a ∼50 kDa EGFP-Hps1 fusion protein band that was absent in the mock-transfected sample (Fig. 2F). Importantly, the expression of EGFP-Hps1 protein decreased upon Ptbp1 and Ptbp1/2 knockdown ∼4 and ∼7 fold, respectively (ANOVA p = 1.4×10^−4^) (Fig. 2F–G). Conversely, the control EGFP protein was expressed at virtually constant levels across all siRNA-treated samples (Fig. 2F–G). Taken together, these results strongly suggest that Hps1 expression levels are controlled by AS-NMD mediated by Ptbp1.

### Hps1 is co-expressed with Ptbp1 *in vivo*

**Figure 3:**
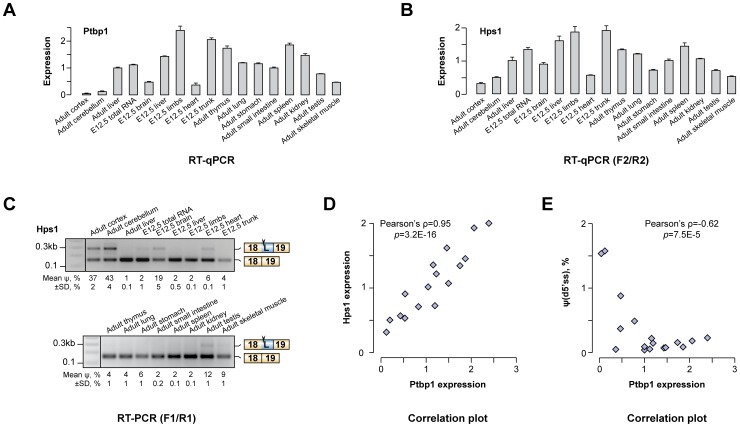
Hps1 is co-expressed with Ptbp1 *in vivo*. (**A**) RT-qPCR analysis of Ptbp1 expression in embryonic (E12.5) and adult mouse tissues. Expression level in adult mouse liver is set to 1. (**B**) RT-qPCR quantitation of Hps1 mRNA expression levels in the same set of mouse tissues as in (A) averaged from three independent experiments ±SD. (**C**) RT-PCR analysis of the Hps1 exon18-exon19 tissue-specific splicing patterns. Relative abundances of the d5′ss-spliced products [ψ(d5′ss)] averaged from two independent experiments ±SD are indicated at the bottom. (**D–E**) Scatter plots showing significant positive correlation between Ptbp1 and Hps1 mRNA expressions and negative correlation between Ptbp1 expression and Hps1 ψ(d5′ss) values.

We wondered if the newly identified AS-NMD regulation could account for Hps1 expression patterns *in vivo*. In line with published reports [28], [46]–[48], our RT-qPCR analyses showed that Ptbp1 was expressed across a wide range of adult and embryonic tissues with the lowest levels observed in brain, heart, skeletal muscle and testis (Fig. 3A). When the same set of tissues was assayed for Hps1 mRNA, we detected a striking positive correlation between Ptbp1 and Hps1 expression levels (Pearson's correlation coefficient ρ = 0.951, p = 3.2×10^−16^; Fig. 3B and Fig. 3D). In addition, both the u5′ss and the d5′ss Hps1 isoforms were detected in brain, heart, skeletal muscle and testis whereas only the u5′ss isoform was present elsewhere (Fig. 3C). Overall, there was a strong negative correlation between Ptbp1 levels and the d5′ss utilization efficiency (Pearson's ρ = −0.626, p = 6.7×10^−4^; Fig. 3E). Similar relationships between Ptbp1 expression and incidence of corresponding splice forms were detected for other newly identified A5C and A3C genes (Fig. S3). Of note, expression patterns between Hps1 and Ptbp2 mRNA levels correlated in a negative fashion (Fig. S4). This argued against a major role of Ptbp2 in shaping Hps1 expression *in vivo* and likely reflected the reciprocal relationship between Ptbp1 and Ptbp2 [28], [47], [49]. We concluded that Ptbp1 but not Ptbp2 may control Hps1 abundance across mouse tissues.

### Polypyrimidine elements between u5′ss and d5′ss of Hps1 exon 18 are necessary for the A5C regulation

**Figure 4:**
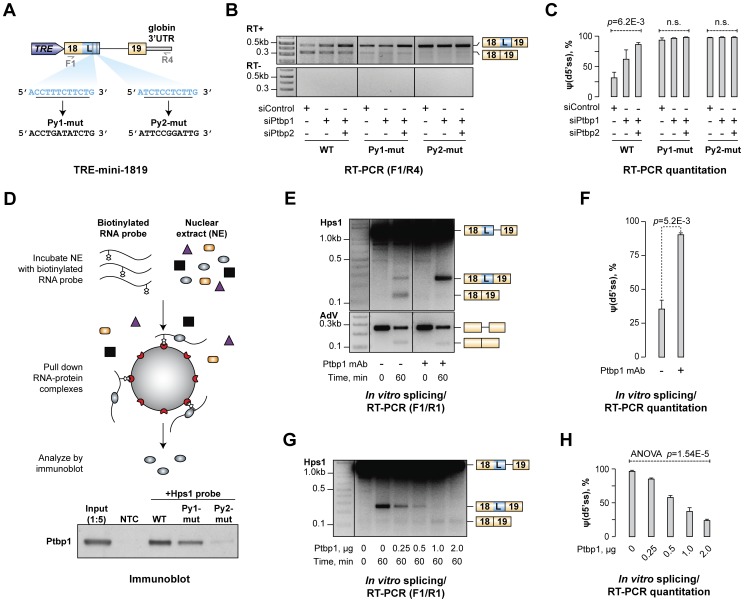
Ptbp1 binds to pyrimidine-rich sequences between u5′ss and d5′ss and directly regulates Hps1 A5C. (**A**) Minigene construct encoding *Hps1* exon 18-intron 18-exon 19 segment. Putative Ptbp1 binding motifs, Py1 and Py2, and their mutated versions are depicted below. (**B**) CAD cells treated with indicated siRNAs were transfected with either the wild-type (WT) TRE-mini-1819 construct or its Py1-mut or Py2-mut derivatives and analyzed by RT-PCR using F1/R4 primers. Considerable accumulation of minigene-specific d5′ss-spliced transcripts in this experiment and Fig. S6B is likely a result of their NMD resistance due to the lack of ORF. (**C**) Quantitation of the relative abundances of the d5′ss-spliced form [ψ(d5′ss)] in (B) averaged from three independent experiments ±SD. (**D**) *Top*, Ptbp1-RNA binding assay. *Bottom*, immunoblot analysis showing readily detectable Ptbp1 interaction with the wild-type mini-1819 RNA, reduced interaction with the Py1-mut mini-1819 RNA and severely diminished interaction with the Py2-mut mini-1819 RNA. NTC is a no-template control. (**E**) Splicing of the Hps1 or AdV RNA substrates was assayed *in vitro* using control-treated or Ptbp1-immunodepleted NEs and the reaction products were analyzed using RT-PCR at the 0- and 60-minute time points. (**F**) Quantitation of the results from (E) showing relative abundance of the d5′ss-spliced form [ψ(d5′ss)] averaged from two independent experiments ±SD. (**G**) Ptbp1-immunodepleted splicing reactions were supplemented with indicated amounts of purified recombinant Ptbp1 protein and the reaction products were analyzed as in (E). (**H**) Relative abundance of the d5′ss-spliced form [ψ(d5′ss)] in (G) averaged from two independent experiments ±SD.

To gain insights into the molecular mechanism underlying Hps1 regulation, we prepared a minigene cassette containing Hps1 exon 18, exon 19 and the intervening intron under control of a doxycycline-inducible promoter [TRE-mini-1819(WT); Fig. 4A]. CAD cells pre-treated with siControl, siPtbp1 or siPtbp1/2 were transfected with this construct and the minigene-specific splicing patterns were analyzed by RT-PCR (Fig. 4B). Similar to the endogenous Hps1 mRNA, minigene-derived transcripts used preferentially u5′ss in the siControl sample and d5′ss in the siPtbp1 and siPtbp1/2 samples (Fig. 4B–C). This indicated that *cis*-elements responsible for the dependence of Hps1 splicing pattern on Ptbp1/2 were located in a vicinity of the regulated exon 18.

Ptbp1 is known to form high-affinity complexes with repeated UCUC, UCUU, CUCU or UUCU motifs [26], [48], [50]. Two pyrimidine-rich stretches (Py1 and Py2) containing consensus tetramers in pyrimidine-rich contexts occur in *Hps1* between the Ptbp1-regulated u5′ss and d5′ss (Fig. 4A) and this arrangement is conserved across mammalian species (Fig. S5). We addressed possible functional significance of these elements by mutating either Py1 or Py2 in the TRE-mini-1819 context (Fig. 4A) and repeating the CAD transfection experiment with the resultant TRE-mini-1819(Py1-mut) and TRE-mini-1819(Py2-mut) constructs. Strikingly, mutation of either of the two Py sequences was sufficient to completely abolish the A5C regulation with the splicing pattern shifting towards d5′ss in siControl, siPtbp1 and siPtbp1/2 samples (Fig. 4B–C).

However, when we assayed splicing of the minigene-encoded transcripts in CAD cells over-expressing comparable amounts of recombinant Ptbp1 or Ptbp2 proteins (Fig. S6A), u5′ss utilization was partially restored in TRE-mini-1819(Py1-mut) and, to a lesser extent, TRE-mini-1819(Py2-mut) (Fig. S6B–C). Notably, recombinant Ptbp1 rescued u5′ss more efficiently than Ptbp2 (Fig. S6B–C) and transcripts derived from the TRE-mini-1819(Py1-mut/Py2-mut) minigene lacking both Py sequences were constitutively spliced at d5′ss both in the control and the Ptbp1- or Ptbp2-over-expressing samples (Fig. S6B–C).

Overall, these experiments suggest that both Py sites are required to orchestrate Hps1 A5C regulation under physiological conditions. Of the two sites, Py2 plays a more decisive role than Py1 and Ptbp1 is a noticeably stronger regulator than Ptbp2.

### Ptbp1 binding to Py1 and Py2 elements regulates the A5C

To test if Ptbp1 directly interacted with Py1 and Py2 sequences, we carried out a biotinylated RNA pull-down assay (Fig. 4D). Interaction between Ptbp1 and a wild-type Hps1 probe comprising both Py1 and Py2 sites was readily detectable by this approach (Fig. 4D). However, mutation of either Py1 or Py2 noticeably reduced this interaction, with a greater reduction in binding affinity observed upon inactivation of Py2 (Fig. 4D and Fig. S6D).

To examine whether Ptbp1 recruitment to the Py sites was responsible for biasing the Hps1 A5C towards u5′ss, we prepared a synthetic RNA comprising the mini-1819(WT) cassette and analyzed splicing of this substrate *in vitro* using HeLa S3 nuclear extract (NE; Fig. 4E). After a 60-min incubation at 30°C, two splicing products were detected by RT-PCR using F1/R1 primers corresponding to splicing at the u5′ss (∼65%) and the d5′ss (∼35%) (Fig. 4E). Notably, when we immunodepleted Ptbp1 from the NE and repeated the experiment, the d5′ss utilization increased to ∼90% (t-test, p = 5.2×10^−3^; Fig. 4E–F). Ptbp1 depletion had no effect on the efficiency of constitutive splicing of a control adenovirus-derived RNA substrate (AdV) (Fig. 4E). To further ensure that the change in the Hps1 splicing upon Ptbp1 withdrawal was a specific effect, we supplemented immunodepleted NE with purified recombinant Ptbp1 protein and repeated the analysis. Notably, the addition of increasing Ptbp1 amounts led to a progressive decline in the d5′ss utilization and a corresponding increase in the u5′ss utilization (Fig. 4G–H). Less efficient u5′ss rescue was observed when we used purified recombinant Ptbp2 instead of Ptbp1 (Fig. S7).

Thus, Ptbp1 binds to the Py1 and Py2 sequences within exon 18/L and directly biases the choice between the two alternate 5′ splice sites towards u5′ss. Similar to our above results, Ptbp2 is less efficient than Ptbp1 in promoting u5′ss utilization *in vitro*.

### Ptbp1 stimulates u5′ss usage

**Figure 5:**
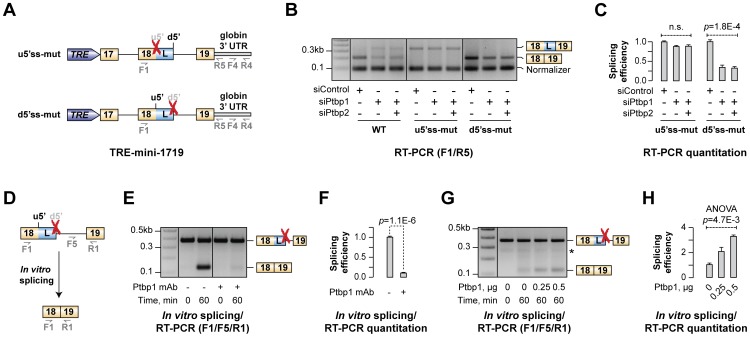
Ptbp1 functions by stimulating u5′ss usage rather than repressing d5′ss. (**A**) TRE-mini-1719 minigene constructs used in this experiment. Red crosses indicate mutational inactivation of the corresponding splice sites. (**B**) CAD cells treated with indicated siRNAs were transfected with either WT or mutated (u5′ss-mut or d5′ss-mut) TRE-mini-1719 constructs and analyzed by multiplex RT-PCR combining two primer pairs, F1/R5 and F4/R4, to detect the ratio between spliced and total minigene-specific transcript levels. (**C**) Relative splicing efficiencies of TRE-mini-1719(u5′ss-mut) the TRE-mini-1719(d5′ss-mut) samples in (B) calculated as a ratio between spliced and total transcript abundance. Data are averaged from three independent experiments ±SD. (**D–E**) Splicing of mini-1819(d5′ss-mut) RNA was assayed in the presence of control-treated or Ptbp1-immunodepleted NE and the reaction products were analyzed by multiplex RT-PCR. (**F**) Quantitation of the results from (**E**), represented as average splicing efficiency from two independent experiments, ±SD. (**G**) Ptbp1-immunodepleted reactions were rescued with increasing concentration of recombinant Ptbp1 protein and analyzed by RT-PCR. (**H**) Quantitation of the results from (G), represented as splicing efficiency averaged from two independent experiments, ±SD.

Two alternative models could account for the above results: (1) direct activation of u5′ss by Ptbp1 or (2) repression of d5′ss indirectly biasing the choice towards u5′ss. To distinguish between these possibilities, we prepared three TRE-mini-1719 minigenes comprising Hps1 exons 17, 18 and 19 along with the intervening introns (Fig. 5A). Of these, TRE-mini-1719(WT) contained intact exon 18 u5′ss and d5′ss and therefore was expected to be regulated similarly to the TRE-mini-1819(WT) minigene above. In the other two constructs, TRE-mini-1719(u5′ss-mut) and TRE-mini-1719(d5′ss-mut), the corresponding sites were inactivated by mutations thus allowing us to test whether Ptbp1/2 had an effect on utilization of the only remaining 5′ss (Fig. 5A).

We introduced these constructs into CAD cells pre-treated with siControl, siPtbp1 or siPtbp1/2 and analyzed the samples 72 hours post-transfection by multiplex RT-PCR using a combination of two primer pairs (F1/R5 and F4/R4) designed to measure the ratio between expression levels of spliced and total minigene-specific transcripts (Fig. 5B). Three distinct RT-PCR products were detectable in TRE-mini-1719(WT) samples with the F4/R4 “normalizer” band at the bottom and the u5′ss- and d5′ss-spliced variants of the F1/R5 amplicon at the top (Fig. 5B). As expected, the ratio between the two top bands changed upon Ptbp1 or Ptbp1/2 knockdown indicating an increased usage of the d5′ss (Fig. 5B). TRE-mini-1719(u5′ss-mut) and TRE-mini-1719(d5′ss-mut) samples gave rise to two products: the F4/R4 normalizer and either the d5′ss- or u5′ss-spliced variant of the F1/R5 amplicon, respectively (Fig. 5B). Importantly, down-regulation of Ptbp1 alone or in combination with Ptbp2 had no detectable effect on the d5′ss-spliced product/normalizer ratio for TRE-mini-1719(u5′ss-mut) but significantly reduced the u5′ss-spliced product/normalized ratio in the TRE-mini-1719(d5′ss-mut) samples (Fig. 5B–C).

Similar results were obtained when we re-analyzed the above samples by RT-qPCR using splice junction-specific primers designed to distinguish between u5′ss and d5′ss use (Fig. S8A–D). Indeed, siPtbp1 and siPtbp1/2 up-regulated the d5′ss-spliced products and diminished the abundance of the u5′ss-spliced ones in the WT, whereas d5′ss utilization was not affected by siPtbp1 and siPtbp1/2 in the u5′ss-mut transcripts. On the other hand, u5′ss was clearly repressed by siPtbp1 and siPtbp1/2 in the d5′ss-mut transcripts. Thus, Ptbp1 appeared to activate the u5′ss rather than repress the d5′ss.

To test whether this could be a direct effect, we carried out an *in vitro* splicing assay with a synthetic mini-1819 RNA substrate mutated at the d5′ss position [mini-1819(d5′ss-mut)] and analyzed the reaction products by RT-PCR (Fig. 5D). As expected, a single u5′ss-derived splice form was detected after a 60-min incubation with HeLa S3 NE (Fig. 5E). The mini-1819(d5′ss-mut) splicing efficiency was dramatically diminished when we immunodepleted Ptbp1 from the NE (∼10-fold down-regulation; t-test p = 1.1×10^−6^; Fig. 5E–F). Analysis of the reaction products by RT-qPCR confirmed that this reduction in splicing efficiency (t-test p = 1.4×10^−6^; Fig. S8H) is accompanied by a reciprocal increase in the pool of unspliced RNA (Fig. S8F). Importantly, supplementing immunodepleted NE with purified recombinant Ptbp1 rescued mini-1819(d5′ss-mut) splicing in a dose-dependent manner (Fig. 5G–H and Fig. S8I). We concluded that Ptbp1 regulates Hps1 A5C by stimulating the u5′ss.

### Regulation of Hps1 AS depends on difference between u5′ss and d5′ss strengths

**Figure 6:**
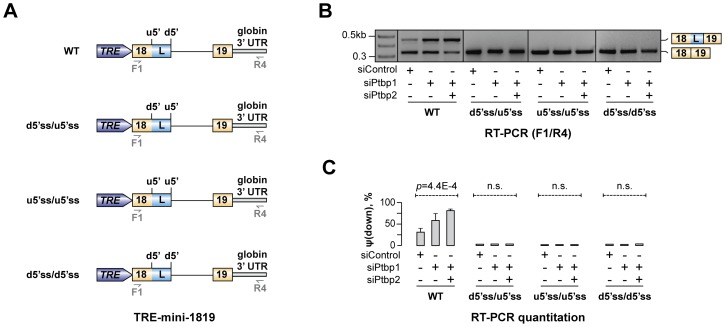
Hps1 regulation depends on u5′ss being weaker than d5′ss. (**A**) TRE-mini-1819 minigenes containing either a wild-type (top) or a permuted arrangement of the two 5′ss. (**B**) CAD cells pre-treated with indicated siRNAs were transfected with the TRE-mini-1819 constructs introduced in (A) and analyzed by RT-PCR using minigene-specific primers F1/R4. Note that the upstream 5′ splice position is constitutively used in all permuted minigene samples. (**C**) Utilization of the topologically downstream 5′ splice site [ψ(down)] in (B) averaged from three independent experiments ±SD.

Our data so far suggested that Ptbp1 interacts with Py1 and Py2 sequences within exon 18 and enhances u5′ss utilization. Interestingly, predicted splicing strength of u5′ss was lower than that of d5′ss (scores S_u5′ss_ = 76.1 vs. S_d5′ss_ = 94.1 obtained using Analyzer Splice Tool server http://ibis.tau.ac.il/ssat/SpliceSiteFrame.htm; [51], [52]) and similar differences were detected in other mammalian species (Table S5). To test if this feature was important for the regulation, we generated a series of modified TRE-mini-1819 minigenes where the natural u5′ss was substituted with the d5′ss or/and the d5′ss was substituted with the u5′ss [TRE-mini-1819(d5′ss/d5′ss), TRE-mini-1819(u5′ss/u5′ss) and TRE-mini-1819(d5′ss/u5′ss); Fig. 6A]. All of these permutations lowered the ΔS = S_d5′ss_-S_u5′ss_ difference between the two 5′ss strengths. Notably, when we transfected CAD cells with the corresponding minigenes, the upstream 5′ splice position was constitutively selected in all siRNA-treated samples (Fig. 6B–C). Similar effects were observed when we weakened the u5′ss or strengthened the d5′ss by substituting them with synthetic 5′ss sequences (Fig. S9). These results are consistent with the model that Hps1 A5C regulation requires u5′ss to be weaker than d5′ss.

### The mechanism regulating Hps1 A5C may recur in other genes

**Figure 7:**
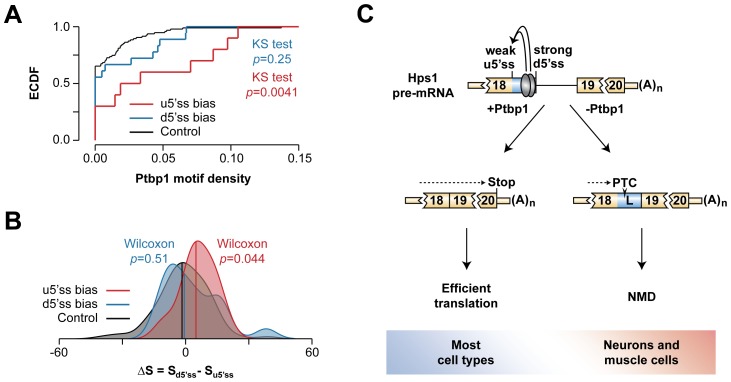
Hps1-like A5C regulation may recur in other genes. (**A**) Empirical cumulative distribution function (ECDF) plot of Ptbp1 motif density within exon segments demarcated by the two 5′ss alternatives in A5C genes. (**B**) Density plot of the difference between d5′ss and u5′ss strengths. Median values are shown by vertical colored lines. (**C**) Model of Ptbp1-dependent AS-NMD regulation of Hps1 expression. Ptbp1 stimulates usage of the intrinsically weak u5′ss thus giving rise to functional Hps1 mRNA. The choice is shifted towards the naturally strong d5′ss in neurons and muscle cells where Ptbp1 is expressed at relatively low levels. This destabilizes Hps1 mRNA through NMD.

We finally asked whether other A5C events uncovered in our bioinformatics screen featured pyrimidine-rich sequences between u5′ss and d5′ss and a weaker u5′ss. To this end, we measured density of putative Ptbp1-binding tetramers (UCUC, UCUU, UUCU, CUCU) between u5′ss and d5′ss in three classes of A5C events: (1) biased towards u5′ss in the presence of Ptbp1, (2) biased towards d5′ss in the presence of Ptbp1 and (3) 100 randomly selected instances of Ptbp1-insensitive A5C (Fig. 7A and Table S6). This analysis showed that the incidence of Ptbp1 motifs was significantly higher in the class 1 events compared to the class 3 control (KS test, p = 0.0041) whereas the class 2 events did not significantly differ from the control (KS test, p = 0.25).

Notably, when we calculated A5C event-specific differences between predicted 5′ss strengths (http://ibis.tau.ac.il/ssat/SpliceSiteFrame.htm; [51], [52]), distribution of the ΔS = S_d5′ss_-S_u5′ss_ values had a significant positive bias for the class 1 (Wilcoxon rank sum test, p = 0.044) but not for the class 2 events (Wilcoxon rank sum test, p = 0.51) (Fig. 7B). Thus, the molecular logic underlying Hps1 regulation might be common amongst A5C events with Ptbp1-induced u5′ss bias.

## Discussion

Mammalian gene expression is extensively controlled at the post-transcriptional level and understanding molecular mechanisms underlying this regulation can generate valuable biomedical insights. In this study, we interrogated transcriptome-wide RNA-seq data and uncovered a number of functionally diverse Ptbp1-dependent A5C and A3C events. We demonstrated that Ptbp1 directly controls the choice between the u5′ss and the d5′ss in Hps1 exon 18 (Fig. 7C). Both Ptbp1 binding motifs (Py1 and Py2) were required for the regulation at physiological Ptbp1 concentrations (Fig. 4A–C) as well as for optimal binding of Ptbp1 to corresponding RNA probes (Fig. 4D and Fig. S6D). Moreover, the splicing switch could be recapitulated by altering Ptbp1 concentration *in vitro* (Fig. 4E–H and Fig. S7). The Ptbp1 paralog Ptbp2 contributed little to the Hps1 A5C control (Fig. S4, Fig. S6A–C and Fig. S7).

Previous work on A5C mechanisms has suggested that recruitment of an hnRNP protein between two alternate 5′ss may bias the AS choice towards the u5′ss by either activating this site directly, repressing its d5′ss competitor or a combination of the two effects [11]–[13]. In the case of Hps1, Ptbp1 appears to achieve this effect by directly stimulating a relatively weak u5′ss rather than repressing its intrinsically stronger d5′ss competitor (Fig. 5, Fig. 6 and Fig. S9). Since A5C targets with Ptbp1-induced u5′ss bias show enrichment of pyrimidine-containing motifs between u5′ss and d5′ss and their u5′ss tends to be weaker than and the d5′ss (Fig. 7A–B), Hps1-like A5C regulation may recur in other genes.

What could be the mechanism allowing Ptbp1 to activate the Hps1 u5′ss? One possibility might involve stimulation of U1 snRNP recruitment to the u5′ss by the Ptbp1 complex assembled at the Py1 and Py2 sequences. Similar strategies have been proposed to mediate activation of an upstream 5′ss by the hnRNP-like protein TIA-1 [13], [53] and other RNA-binding proteins interacting with downstream intronic splicing enhancers [9], [54]. Moreover, Ptbp1 complex assembled on the c-Src pre-mRNA in the vicinity of the AS exon N1 has been shown to form contacts with U1 recruited to the N1 5′ss [55]. Although this leads to repression of N1 splicing, interaction between Ptbp1 and U1 might result in opposite effects in other AS contexts with distinct structures of the ternary complex between Ptbp1, pre-mRNA and U1. Similar to position-specific effects on AS observed for other RNA-binding proteins [56]–[58], Ptbp1 tends to function as a splicing repressor when recruited upstream or/and within AS exons and as an activator when bound to downstream sequences [48], [59]. Additional characterization of the Hps1 A5C may shed new light on this poorly understood phenomenon.

Intriguingly, functionality of the Hps1 A5C appears to rely on a finely tuned balance between the u5′ss and d5′ss strengths, since all mutations strengthening the relatively weak u5′ss or weakening the relatively strong d5′ss lead to constitutive utilization of the u5′ss (Fig. 6 and Fig. S9). It is somewhat surprising that recombinant Hps1 transcripts containing two equally strong or equally weak 5′ss fail to generate a mixture of the two splice isoforms upon Ptbp1 and Ptbp2 withdrawal. This might hint at the existence of additional factors biasing the A5C towards the u5′ss. Interestingly, u5′ss rescue by purified Ptbp1 in Ptbp1-depleted *in vitro* splicing reactions was incomplete in a subset of our assays (e.g., compare Fig. 5E and Fig. 5G). This would be consistent with direct interaction of a hypothetical u5′ss-stimulating factor with Ptbp1 protein. We plan to address this interesting prediction in our future studies.

The AS-NMD circuitry identified in our work (Fig. 7C) may account for tissue-specific *Hps1* expression. We show that splicing at the u5′ss gives rise to functional Hps1 ORF whereas utilization of the d5′ss generates NMD-susceptible transcripts (Fig. 2). Since Ptbp1 is required for selecting the u5′ss alternative, this mechanism likely ensures a strong positive correlation between Hps1 and Ptbp1 expression levels across tissues (Fig. 3). Type 1 HPS caused by homozygous loss-of-function mutations in *Hps1* is typically manifested by reduced pigmentation, prolonged bleeding and lysosomal storage defects in many tissues. Further complications include inflammatory bowel disease and life-limiting pulmonary fibrosis [32]–[34], [44]. Despite the multi-organ nature of this syndrome, HPS patients and *pale ear* mice do not typically develop neurological, cardiac or muscular problems [32]–[34], [44], [60]. This would be consistent with the naturally low Hps1 levels in tissues expressing little Ptbp1.

Ptbp1 has been previously shown to regulate expression levels of several genes through AS coupled with NMD or nuclear retention and elimination (NRE) of aberrantly spliced transcripts [28]–[30]. However, in all of these cases Ptbp1 down-regulation increased steady-state levels of the corresponding mRNAs in the neuronal lineage. Thus, Hps1 provides a remarkable example of AS-NMD circuitry enabling tight co-expression of a target gene and its post-transcriptional master regulator. One possible advantage of this strategy could be “de-noising” of the Hps1 expression outputs in the presence of Ptbp1, since Ptbp1 own expression is stabilized by an auto-regulatory AS-NMD feedback loop [27]. On the other hand, this may allow developmental dynamics of *Hps1* to be synchronized with expression changes in other Ptbp1 targets thus maximizing the overall coordination of cellular differentiation process.

In conclusion, our work uncovers a large set of Ptbp1-controlled A5C and A3C events and provides molecular insights into mechanism regulating expression output of the disease-related *Hps1* gene. We predict that further examples of the master regulator tracking strategy described here for Hps1 will be identified in the future.

## Materials and Methods

### Plasmids

pGEM3Zf(+) and pEGFP-C1 vectors were from Promega and Clontech, respectively. AdML-M3 construct encoding an adenovirus-specific splicing substrate (Addgene #11244) and pEM275 and pEM288 plasmids encoding FLAG-tagged Ptbp1 and Ptbp2, respectively, were described previously [28], [61]. New constructs were generated using standard molecular cloning techniques and enzymes from NEB as outlined in Table S7. Site-specific mutagenesis was done using KAPA HiFi DNA polymerase (KAPA Biosystems) and corresponding mutagenic primers (Table S8). All plasmid maps and sequences are available on request.

### Cell cultures

CAD cells (Cath.a-derived mouse neuroblastoma) [62] were cultured in Dulbecco's Modified Eagle Medium/High Glucose (DMEM; GIBCO, USA), supplemented with 11% FetalClone III Serum (Hyclone, USA), 1 mM sodium pyruvate (GIBCO, USA), 100 IU/ml penicillin and 100 µg/ml streptomycin, at 37°C in the presence of 5% CO_2_. For transfection experiments, cells were plated in the CAD medium without antibiotics at a density of 4×10^5^ cells per well of a tissue culture 6-well plate. Twelve hours post-plating, cells were transfected with corresponding siRNAs (ThermoScientific Dharmacon, USA) using Lipofectamine RNAiMAX (Invitrogen, USA). Following 36-hour incubation, cell cultures were typically re-transfected with 1 µg of a minigene plasmid using Lipofectamine 2000 and incubated for another 36 hours prior to RNA harvest. In some experiments, cells were treated with either 100 µg/ml of CHX dissolved in DMSO or DMSO control for 8 hours. In the FLAG-Ptbp1 and FLAG-Ptbp2 over-expression experiments, 35 ng of pEM275 or 90 ng of pEM288 was co-transfected with 100 ng of Hps1 TRE-mini-1819 minigene and the total DNA amount was adjusted to 1 µg with an EGFP-encoding control plasmid (pCIG) and incubated for 48 hours.

### RT-PCR and RT-qPCR

Total RNA was harvested from adherent cells using Trizol (Invitrogen). RNA was subsequently treated with 50 units/ml of RQ1 DNase (Promega) at 37°C for 1 hour to eliminate traces of genomic DNA. First-strand cDNA synthesis (RT) was typically performed in 10 µl reactions containing 2.5 µg of total RNA, 50 pmol of a random decamer primer (N10), 40 units of rRNAsin (Promega) and 100 units of SuperScript III reverse transcriptase (Invitrogen) at 50°C for 1 hour. Regular PCRs were carried out using Taq DNA polymerase (KAPA Biosystems) and amplification products were resolved by gel electrophoresis in 2% or 3.5% agarose gels. Quantitative PCR (qPCR) assays were done in triplicate using SYBR FAST qPCR Master Mix (KAPA Biosystems) and a StepOnePlus real-time PCR system (Applied Biosystems) and the signals were normalized to Gapdh mRNA levels. Relevant primer sequences are provided in Table S7.

### Biotinylated RNA pull-down assays

RNA probes were generated by transcribing linearized plasmid DNA *in vitro* with T7 polymerase (Promega) and biotin RNA labeling mix (Roche) for 2 hours at 37°C. Reactions were stopped by adding 1 unit of RQ1 DNase per 1 µg of template DNA and incubating the mixtures at 37°C for 15 min. Biotinylated RNAs were then extracted using phenol-chloroform (1∶1) mixture, precipitated with ethanol and resuspended in DEPC-treated water. Pull-down assays were carried out by incubating 2 µg of purified RNA probes in 20 µl of buffer D (20 mM HEPES, pH 7.9, 100 mM KCl, 20% Glycerol, 0.5 mM DTT and 0.2 mM EDTA) supplemented with 80 ng yeast tRNA, 2.5 µg heparin, 40 units of rRNAsin (Promega) and 50% HeLa S3 NE (vol/vol; dialyzed against buffer D; ∼100 µg protein in total) for 30 min at room temperature. RNA-protein complexes were then incubated with 20 µl of Streptavidin Sepharose Beads (Sigma) pre-washed in buffer D for 1 hour at 4°C. The beads were then washed thrice with buffer D and the RNA-associated proteins were eluted by boiling the beads for 10 min in 30 µl of 1× SDS PAGE sample buffer (0.0625 M Tris-HCl pH 6.8, 2% SDS, 5% β-mercapthoethanol, 10% glycerol and 0.01% bromophenol blue) and subsequently analyzed by immunoblotting.

### *In vitro* splicing assay

Splicing reactions (20 µl) contained 200 ng of unlabeled splicing substrate RNA prepared by *in vitro* transcription [29], 2 µl of 10× splicing reaction buffer (120 mM HEPES, pH 7.9, 32 mM MgCl_2_ and 725 mM KCl), 1 mM ATP (NEB), 20 mM phosphocreatine (Sigma) 2.5% poly(vinyl alcohol) (Sigma, MW 30–70K), 1 mM DTT, 10 units of rRNAsin (Promega) and 30% HeLa S3 NE (vol/vol; ∼60 µg protein; dialyzed against buffer D). Following incubation at 30°C for 60 min the reactions were stopped by the addition of 200 µl of PK buffer (10 mM Tris-HCl pH 7.4, 1% SDS, 150 mM NaCl and 10 mM EDTA) and 0.25 mg/ml proteinase K (Fermentas) and incubated for another 15 min at 37°C. Splice products were extracted with phenol-chloroform (1∶1), precipitated with ethanol, dissolved in DEPC-treated water (Invitrogen) and analyzed by RT-PCR using corresponding primer pairs (Table S7). Adenovirus-specific splicing was assayed using EMO2619/2622 primers (Table S7). In some experiments, NE was immunodepleted for Ptbp1. For this purpose, 40 µg of mouse monoclonal anti-PTBP antibody (Invitrogen, clone 1) was incubated with 40 µl of protein G Sepharose beads (GE Healthcare) overnight at 4°C with continuous rotation. Beads were subsequently washed thrice with buffer D and incubated with 50 µl of HeLa S3 NE for another 4 h at 4°C with rotation. Ptbp1-depleted NE was then recovered by pelleting the beads at 3,000 rpm for 2 min.

### Immunoblotting

Proteins were extracted from PBS-washed adherent cells using NP40 buffer [20 mM Tri-HCl, pH 7.5, 150 mM NaCl, 5 mM EDTA, 10% glycerol, 1% Nonidet P-40, 1 mM phenylmethanesulfonyl fluoride and recommended concentration of cOmplete EDTA-free protease inhibitor cocktail (Roche; one tablet per 50 ml)] and quantified using a BCA protein assay kit (Thermo Scientific). Proteins were then separated by 4–20% gradient SDS-PAGE (Bio-Rad), electrotransferred to nitrocellulose membranes and analyzed using the following primary antibodies: mouse monoclonal anti-Ptbp1 (1∶1000, Invitrogen), mouse monoclonal anti-Ptbp2 (1∶20000; a gift from R. Darnell), mouse monoclonal anti-GFP (1∶2000, Invitrogen), mouse monoclonal anti-FLAG M2 (1∶1000, Sigma), mouse monoclonal anti-Gapdh (1∶10000, Ambion). Immunoblot signals were visualized using corresponding secondary antibodies conjugated with horseradish peroxidase (GE Healthcare) and Immobilon Western ECL reagents (Milipore).

### Bioinformatics

To identify A5C and A3C events, fastq RNA-seq files for CAD cells treated with siControl, siPtbp1 or siPtbp1/2 ([29]; NCBI Gene Expression Omnibus accession number GSE37933) were analyzed using TopHat aligner [63] and mm9 mouse genome assembly. The aligned junction read files were then processed using in-house Perl scripts (Dataset S1) designed to identify all possible pairs for A5C (u5′ss-c3′ss and d5′ss-c3′ss) and A3C (c5′ss-u3′ss and c5′ss-d3′ss) junctions across experimental samples. Junction reads corresponding to cassette exons were depleted by requesting that u5′ss in A5C and d3′ss in A3C pairs map to a known exon present in the UCSC gene, RefSeq gene or mRNA libraries (http://genome.ucsc.edu/). A5C and A3C pairs undergoing significant changes were identified by Fisher's exact test using R (http://CRAN.R-project.org/doc/FAQ/R-FAQ.html) (Dataset S1).

### Ethics statement

All mouse work was conducted according to protocol approved by the Institutional Animal Care and Use Committee (IACUC) of Nanyang Technological University, Singapore. No surviving procedures were used. Mice were euthanized using isoflurane overdose procedure as recommended by IACUC.

## Supporting Information

Figure S1 Optimization of the A5C and A3C discovery pipeline and validation of newly identified events. (**A**) To make sure our A5C/A3C analysis pipeline performed adequately, we first analyzed training RNA-seq data from CAD and L929 cells expected to exhibit markedly different AS patterns. This uncovered 195 A5C and 171 A3C significant cell line-specific events (p<0.05, Fisher's exact test) with apparent differences in the isoform-specific percent spliced in statistic (ψ [7]) exceeding 5% (Tables S1 and S2). Satisfyingly, all 8 examples selected for reverse transcriptase (RT)-PCR validation showed readily detectable differences in spicing patterns between the two cell lines. (**B**) RT-PCR validation of a subset of Ptbp1-regulated A5C and A3C events. Other examples are presented in Fig. 1D. ψ values show the abundance of the longer splice form as a percentage of the total averaged from 3 experiments.
(TIF)

Figure S2 Ptbp1 regulates Hps1 mRNA abundance through AS-NMD. (**A**) Immunoblot analysis of CAD cells treated as in Fig. 2B with Ptbp1- and Ptbp2-specific antibodies. Gapdh-specific antibody was used as a lane loading control. (**B**) CAD cells pre-treated with siControl, siPtbp1 or siPtbp1/2 were transfected with siUpf1 or siControl and the Hps1 splicing pattern was analyzed by RT-PCR with F1/R1 primers. (**C**) Relative utilization of the d5′ss form in (B). (**D**) RT-qPCR quantitation of the Hps1 and Upf1 expression in CAD samples treated as in (B). (**E**) Hps1-EGFP-specific A5C patterns in samples introduced in Fig. 2E were analyzed by RT-PCR with F6/R1 primers.
(TIF)

Figure S3 Ptbp1-dependent A5C and A3C events are regulated in a tissue-specific manner. AS patterns of indicated mRNAs in adult mouse liver, cerebellum and cortex were analyzed by RT-PCR. Note that tissue-specific splice form preferences are consistent with relatively high expression of endogenous Ptbp1 in liver and low expression in brain (see Fig. 3A). ψ values for the abundance of the longer isoform are averaged from 3 experiments.
(TIF)

Figure S4 Tissue-specific patterns of Ptbp2 expression. (**A**) RT-qPCR analysis of Ptbp2 expression in embryonic (E12.5) and adult mouse tissues. Expression level in adult mouse liver is set to 1. Data are averaged from three independent experiments ±SD. (**B**) Scatter plot showing a modest but significant negative correlation between Hps1 and Ptbp2.
(TIF)

Figure S5 Conservation of the Hps1 exon 18/L cis-elements across mammals. Sequences labeled in red are consensus Ptbp1-binding motifs occurring within pyrimidine-rich contexts, Py1 and Py2. Also shown are the u5′ss and the d5′ss as well as the premature termination codon (PTC).
(TIF)

Figure S6 Contribution of the Py1 and Py2 sequences to the Hps1 A5C regulation. (**A**) Immunoblot analysis showing that CAD cells transfected with optimized plasmid blends (see Materials and Methods) express comparable amounts of FLAG-tagged Ptbp1 and Ptbp2. (**B**) CAD cells expressing either control or FLAG-Ptbp1- or FLAG-Ptbp2-encoding constructs as in (A) were co-transfected with indicated TRE-mini-1819 minigenes and analyzed by RT-PCR using F1/R4 primers. (**C**) Quantitation of the results in (B). (**D**) Quantitation of the relative amount of Ptbp1 bound to Hps1 RNA probes as described in Fig. 4D. Data in (C and D) are averaged from three independent experiments ±SD.
(TIF)

Figure S7 Ptbp1 is more efficient than Ptbp2 in regulating the Hps1 A5C *in vitro*. (**A**) Coomassie-stained SDS-PAGE analysis of purified recombinant Ptbp1 and Ptbp2 proteins. (**B**) The effect of increasing amounts of recombinant Ptbp1 and Ptbp2 on *in vitro* splicing of a wild-type Hps1 RNA substrate in Ptbp1-immunodepleted NE. (**C**) Quantitation of the data in (B) showing significantly stronger down-regulation of the d5′ss-spliced products in reactions containing recombinant Ptbp1 as compared to those supplemented with Ptbp2. Data are averaged from two independent experiments ±SD.
(TIF)

Figure S8 Quantitative analyses of Hps1 A5C *in vivo* and *in vitro*. (**A–D**) RT-qPCR quantitation of (A–B) u5′ss-spliced and (C–D) d5′ss-spliced products for indicated TRE-mini-1719 minigenes expressed in CAD cells as outlined in Fig. 5B. Note that siPtbp1 and siPtbp2 reduce u5′ss utilization in TRE-mini-1719(d5′ss-mut) samples but have no detectable effect on d5′ss utilization in TRE-mini-1719(d5′ss-mut) samples. (**E–F**) RT-qPCR quantitation of residual unspliced mini-1819(d5′ss-mut) RNA substrate after incubating it for 60 minutes with control- or Ptbp1-depleted NEs. Note that significantly more mini-1819(d5′ss-mut) RNA substrate remains unspliced in the Ptbp1-depleted samples. (**G–I**) RT-qPCR quantitation of spliced products for experiments described in Fig. 5E and Fig. 5G, respectively. Data in (B, D, F, H and I) are averaged from three independent experiments ±SD.
(TIF)

Figure S9 Hps1 regulation depends on u5′ss being weaker than d5′ss. (**A**) TRE-mini-1819 minigenes with mutated u5′ss or d5′ss. (**B**) Strengths of the wild-type and mutant 5′ss sequences predicted by Analyzer Splice Tool (http://ibis.tau.ac.il/ssat/SpliceSiteFrame.htm; [51], [52]) (**C**) siRNA-treated CAD cells were transfected with the TRE-mini-1819 constructs introduced in (A) and analyzed by RT-PCR using minigene-specific primers F1/R4. (**D**) Usage of topologically downstream splice site [ψ(down)] in (C) averaged from two independent experiments ±SD.
(TIF)

Table S1 A5C events differentially regulated between CAD and L929 cells.
(XLSX)

Table S2 A3C events differentially regulated between CAD and L929 cells.
(XLSX)

Table S3 A5C events regulated by Ptbp1 in CAD cells.
(XLSX)

Table S4 A3C events regulated by Ptbp1 in CAD cells.
(XLSX)

Table S5 Difference between u5′ss and d5′ss strengths in mammalian *Hps1* genes.
(XLSX)

Table S6 List of class 1, class 2 and class 3 control genes used in Fig. 7A–B.
(XLSX)

Table S7 Plasmids generated in this study.
(XLSX)

Table S8 Primers used in this study.
(XLSX)

Dataset S1 RNA-seq data analysis pipeline for identifying significantly regulated A5C and A3C events.
(ZIP)
