# Supplementary figures and images for: Regulation of mRNA Abundance by Polypyrimidine Tract-Binding Protein-Controlled Alternate 5′ Splice Site Choice
Source: PLoS Genet. 2014 Nov 6;10(11):e1004771. doi: 10.1371/journal.pgen.1004771 (PMC4222953; doi:10.1371/journal.pgen.1004771)

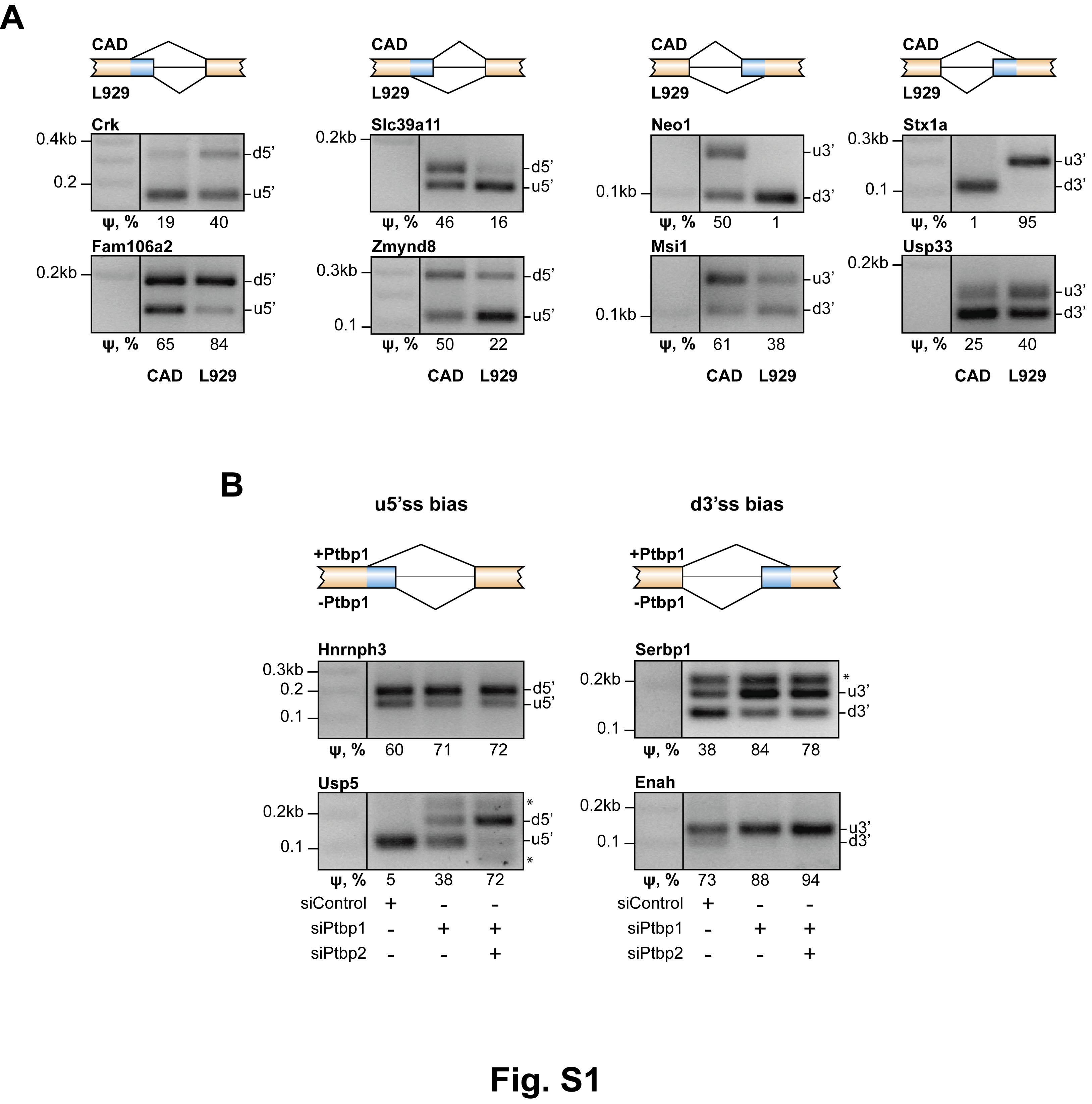

Supplement: Figure S1 — Optimization of the A5C and A3C discovery pipeline and validation of newly identified events. (A) To make sure our A5C/A3C analysis pipeline performed adequately, we first analyzed training RNA-seq data from CAD and L929 cells expected to exhibit markedly different AS patterns. This uncovered 195 A5C and 171 A3C significant cell line-specific events (p<0.05, Fisher's exact test) with apparent differences in the isoform-specific percent spliced in statistic (ψ [7]) exceeding 5% (Tables S1 and S2). Satisfyingly, all 8 examples selected for reverse transcriptase (RT)-PCR validation showed readily detectable differences in spicing patterns between the two cell lines. (B) RT-PCR validation of a subset of Ptbp1-regulated A5C and A3C events. Other examples are presented in Fig. 1D. ψ values show the abundance of the longer splice form as a percentage of the total averaged from 3 experiments. (TIF) [file pgen.1004771.s001.tif]

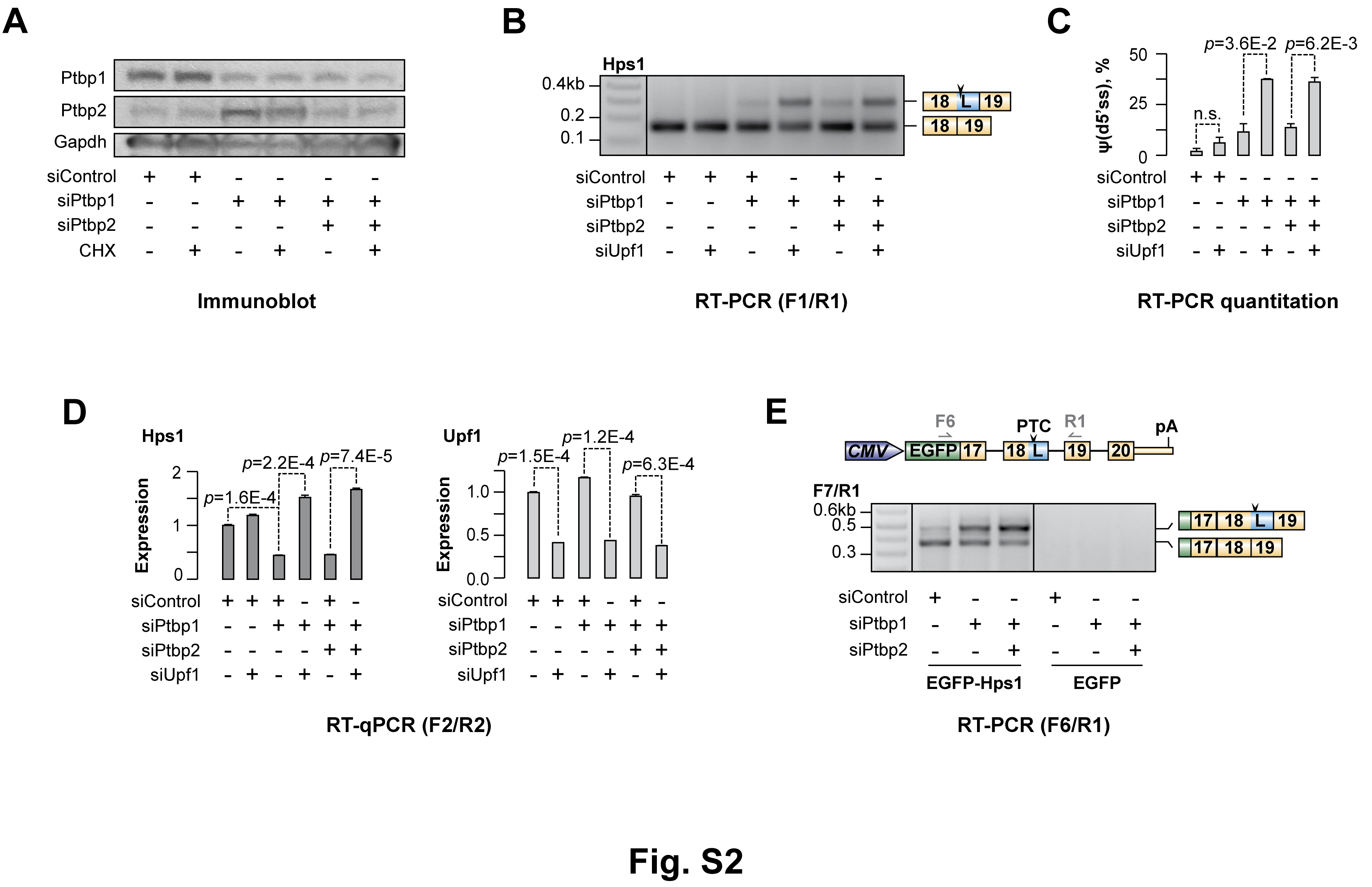

Supplement: Figure S2 — Ptbp1 regulates Hps1 mRNA abundance through AS-NMD. (A) Immunoblot analysis of CAD cells treated as in Fig. 2B with Ptbp1- and Ptbp2-specific antibodies. Gapdh-specific antibody was used as a lane loading control. (B) CAD cells pre-treated with siControl, siPtbp1 or siPtbp1/2 were transfected with siUpf1 or siControl and the Hps1 splicing pattern was analyzed by RT-PCR with F1/R1 primers. (C) Relative utilization of the d5′ss form in (B). (D) RT-qPCR quantitation of the Hps1 and Upf1 expression in CAD samples treated as in (B). (E) Hps1-EGFP-specific A5C patterns in samples introduced in Fig. 2E were analyzed by RT-PCR with F6/R1 primers. (TIF) [file pgen.1004771.s002.tif]

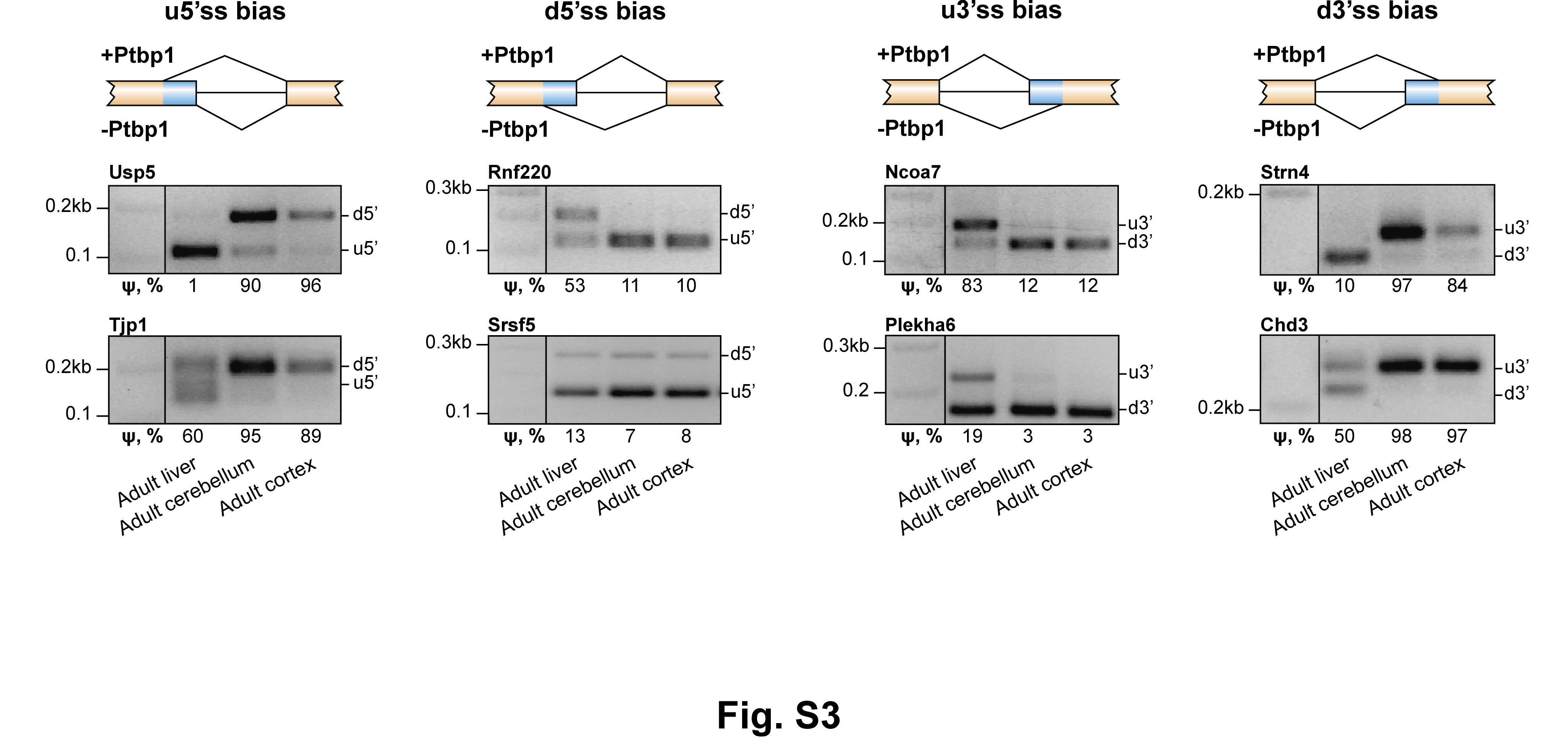

Supplement: Figure S3 — Ptbp1-dependent A5C and A3C events are regulated in a tissue-specific manner. AS patterns of indicated mRNAs in adult mouse liver, cerebellum and cortex were analyzed by RT-PCR. Note that tissue-specific splice form preferences are consistent with relatively high expression of endogenous Ptbp1 in liver and low expression in brain (see Fig. 3A). ψ values for the abundance of the longer isoform are averaged from 3 experiments. (TIF) [file pgen.1004771.s003.tif]

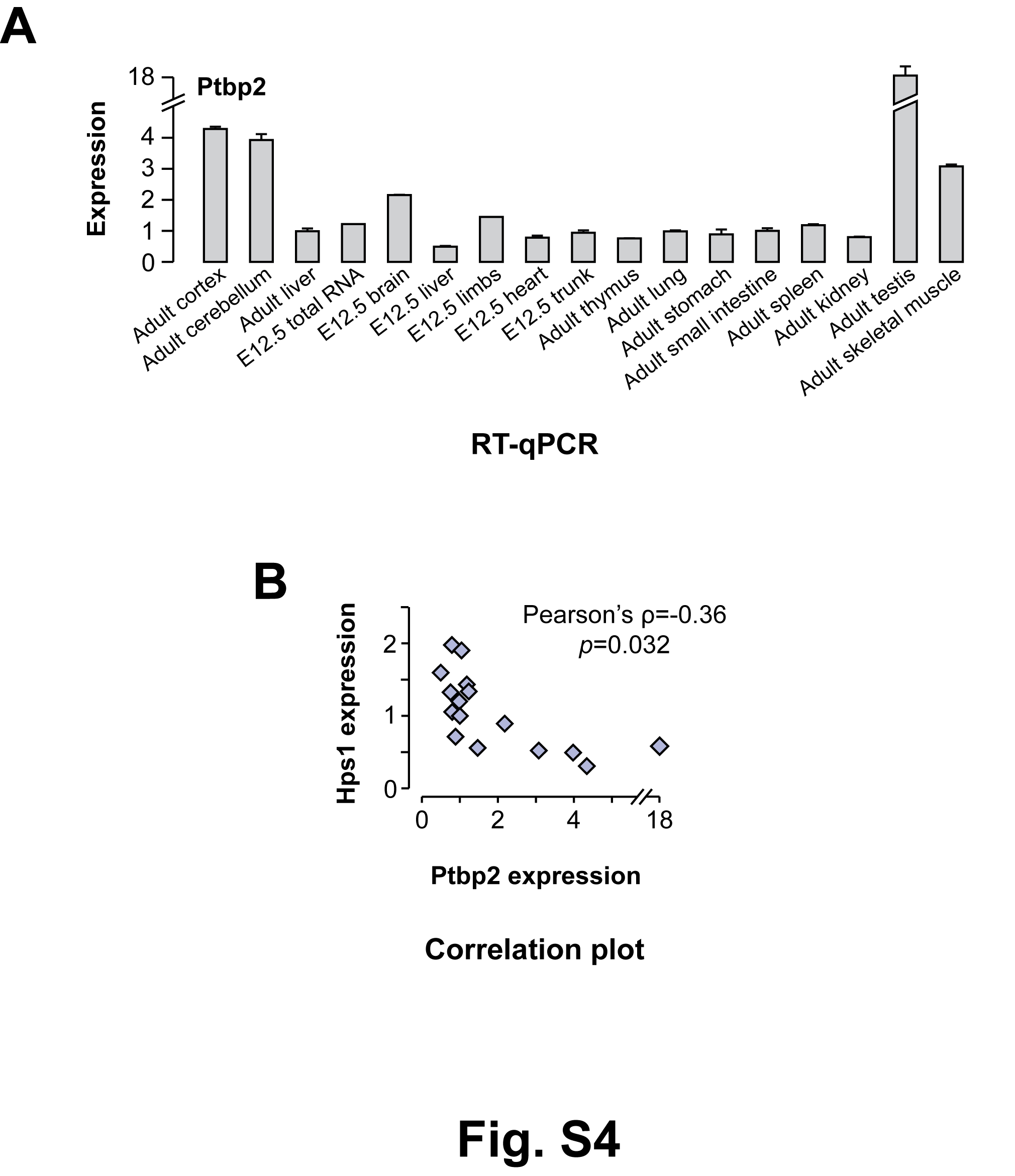

Supplement: Figure S4 — Tissue-specific patterns of Ptbp2 expression. (A) RT-qPCR analysis of Ptbp2 expression in embryonic (E12.5) and adult mouse tissues. Expression level in adult mouse liver is set to 1. Data are averaged from three independent experiments ±SD. (B) Scatter plot showing a modest but significant negative correlation between Hps1 and Ptbp2. (TIF) [file pgen.1004771.s004.tif]

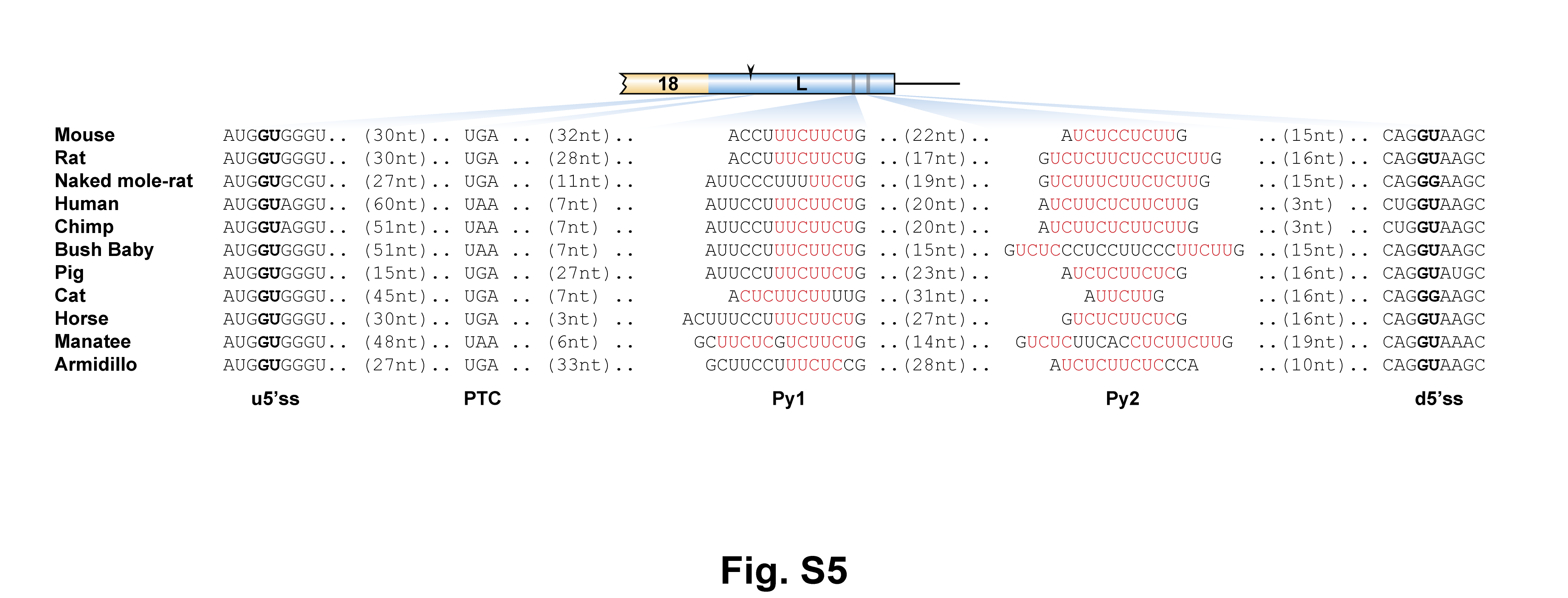

Supplement: Figure S5 — Conservation of the Hps1 exon 18/L cis-elements across mammals. Sequences labeled in red are consensus Ptbp1-binding motifs occurring within pyrimidine-rich contexts, Py1 and Py2. Also shown are the u5′ss and the d5′ss as well as the premature termination codon (PTC). (TIF) [file pgen.1004771.s005.tif]

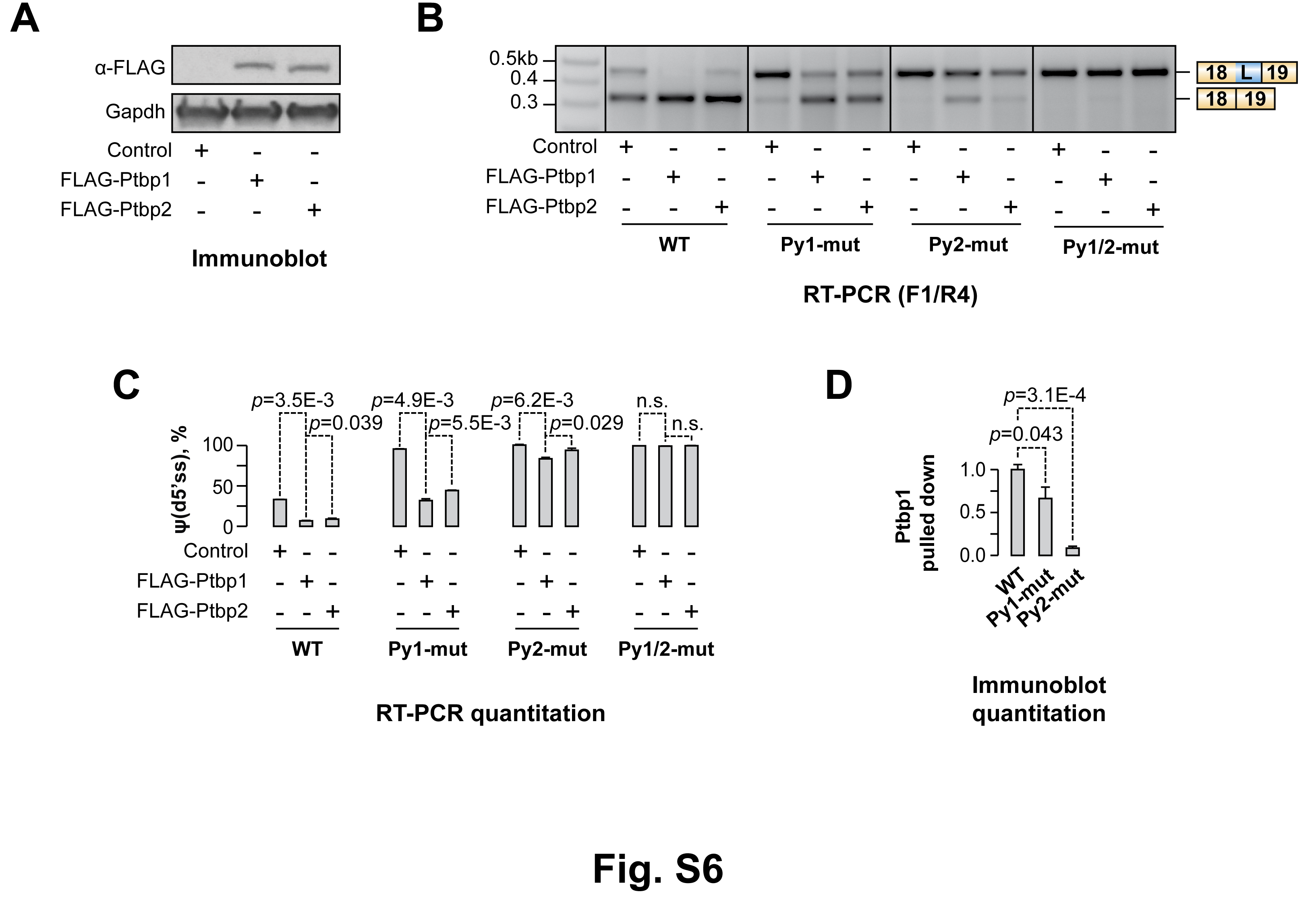

Supplement: Figure S6 — Contribution of the Py1 and Py2 sequences to the Hps1 A5C regulation. (A) Immunoblot analysis showing that CAD cells transfected with optimized plasmid blends (see Materials and Methods) express comparable amounts of FLAG-tagged Ptbp1 and Ptbp2. (B) CAD cells expressing either control or FLAG-Ptbp1- or FLAG-Ptbp2-encoding constructs as in (A) were co-transfected with indicated TRE-mini-1819 minigenes and analyzed by RT-PCR using F1/R4 primers. (C) Quantitation of the results in (B). (D) Quantitation of the relative amount of Ptbp1 bound to Hps1 RNA probes as described in Fig. 4D. Data in (C and D) are averaged from three independent experiments ±SD. (TIF) [file pgen.1004771.s006.tif]

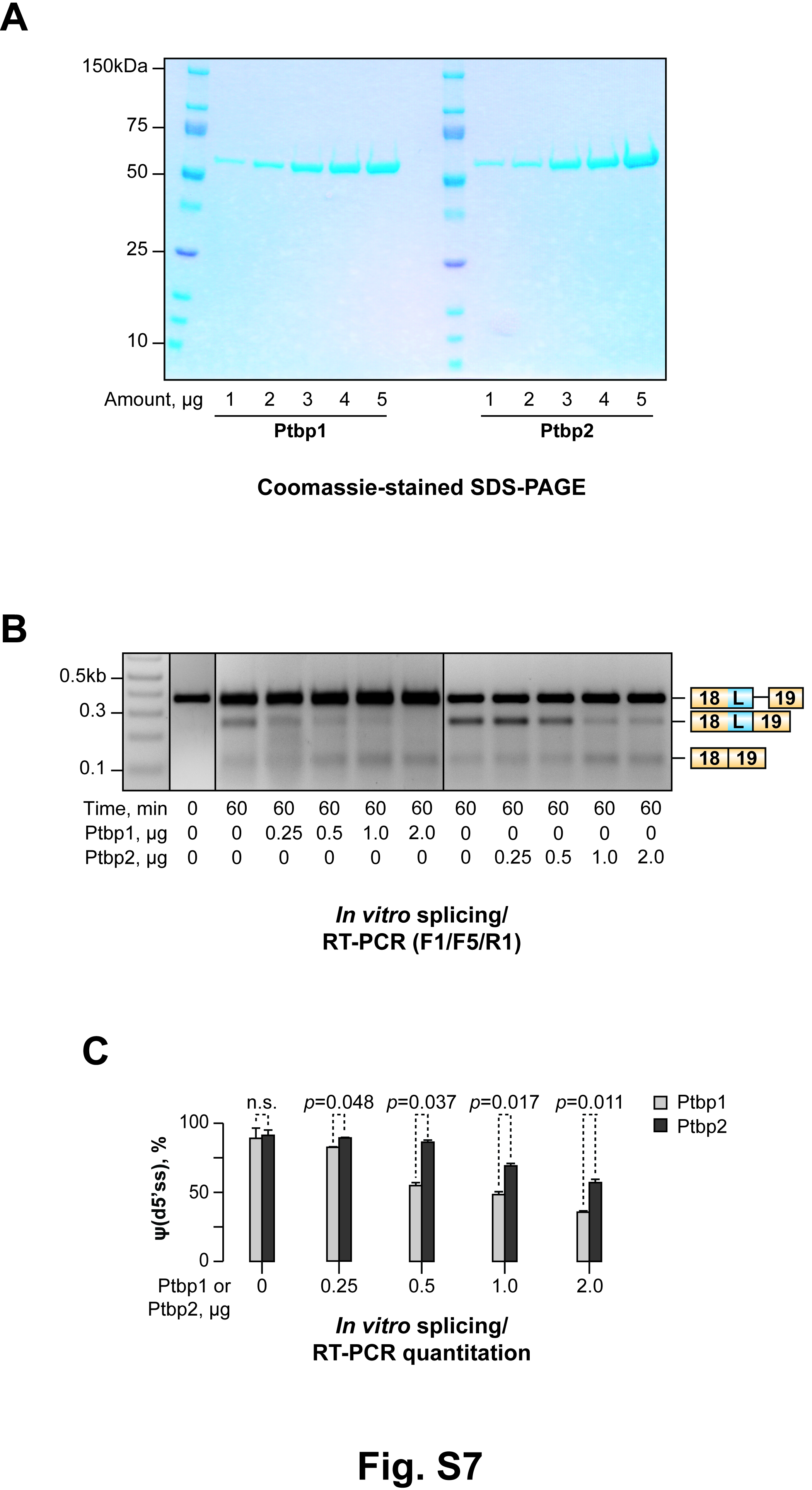

Supplement: Figure S7 — Ptbp1 is more efficient than Ptbp2 in regulating the Hps1 A5C in vitro. (A) Coomassie-stained SDS-PAGE analysis of purified recombinant Ptbp1 and Ptbp2 proteins. (B) The effect of increasing amounts of recombinant Ptbp1 and Ptbp2 on in vitro splicing of a wild-type Hps1 RNA substrate in Ptbp1-immunodepleted NE. (C) Quantitation of the data in (B) showing significantly stronger down-regulation of the d5′ss-spliced products in reactions containing recombinant Ptbp1 as compared to those supplemented with Ptbp2. Data are averaged from two independent experiments ±SD. (TIF) [file pgen.1004771.s007.tif]

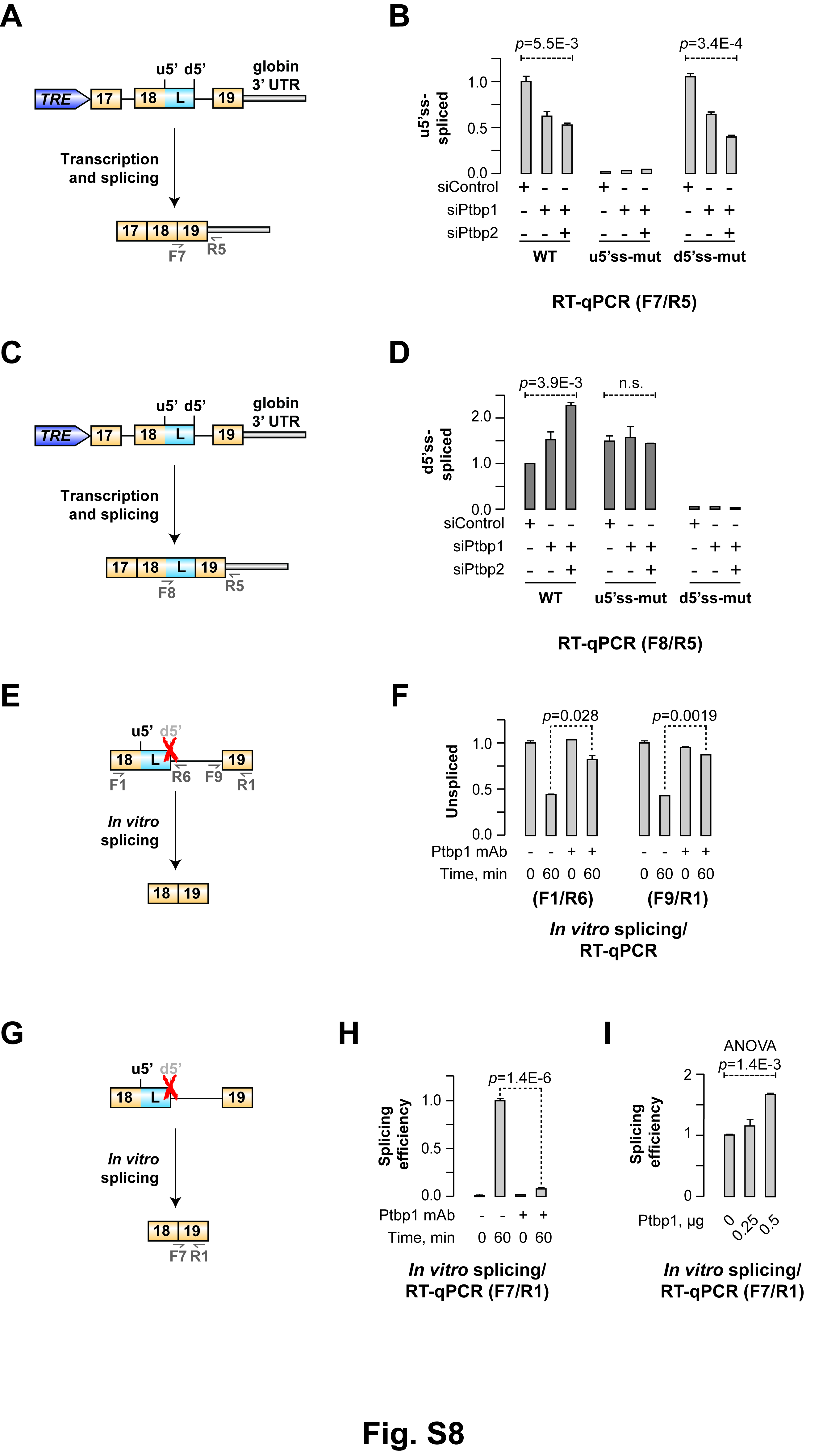

Supplement: Figure S8 — Quantitative analyses of Hps1 A5C in vivo and in vitro. (A–D) RT-qPCR quantitation of (A–B) u5′ss-spliced and (C–D) d5′ss-spliced products for indicated TRE-mini-1719 minigenes expressed in CAD cells as outlined in Fig. 5B. Note that siPtbp1 and siPtbp2 reduce u5′ss utilization in TRE-mini-1719(d5′ss-mut) samples but have no detectable effect on d5′ss utilization in TRE-mini-1719(d5′ss-mut) samples. (E–F) RT-qPCR quantitation of residual unspliced mini-1819(d5′ss-mut) RNA substrate after incubating it for 60 minutes with control- or Ptbp1-depleted NEs. Note that significantly more mini-1819(d5′ss-mut) RNA substrate remains unspliced in the Ptbp1-depleted samples. (G–I) RT-qPCR quantitation of spliced products for experiments described in Fig. 5E and Fig. 5G, respectively. Data in (B, D, F, H and I) are averaged from three independent experiments ±SD. (TIF) [file pgen.1004771.s008.tif]

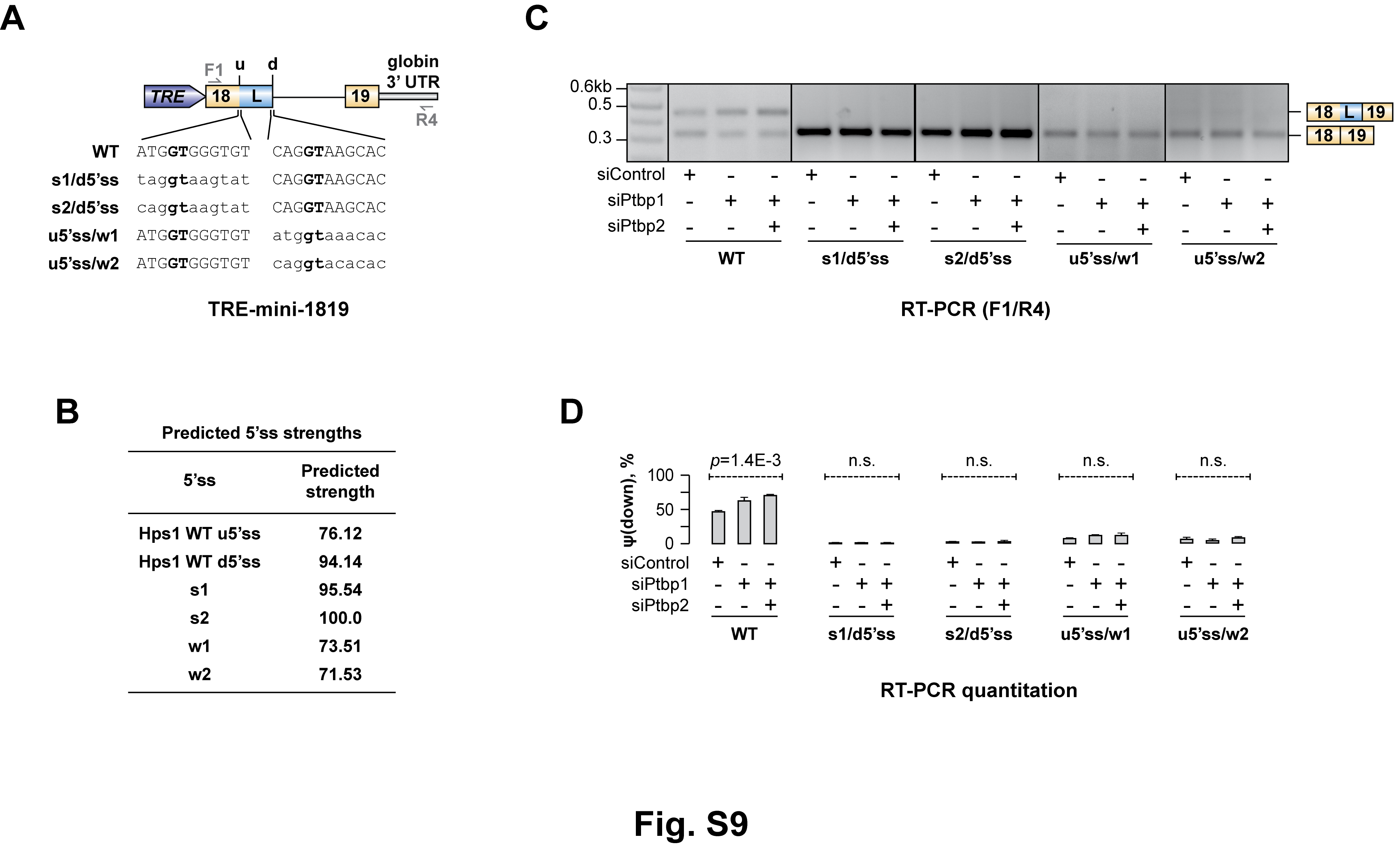

Supplement: Figure S9 — Hps1 regulation depends on u5′ss being weaker than d5′ss. (A) TRE-mini-1819 minigenes with mutated u5′ss or d5′ss. (B) Strengths of the wild-type and mutant 5′ss sequences predicted by Analyzer Splice Tool (http://ibis.tau.ac.il/ssat/SpliceSiteFrame.htm; [51], [52]) (C) siRNA-treated CAD cells were transfected with the TRE-mini-1819 constructs introduced in (A) and analyzed by RT-PCR using minigene-specific primers F1/R4. (D) Usage of topologically downstream splice site [ψ(down)] in (C) averaged from two independent experiments ±SD. (TIF) [file pgen.1004771.s009.tif]
