# Supplementary material for: Regulation of mRNA Abundance by Polypyrimidine Tract-Binding Protein-Controlled Alternate 5′ Splice Site Choice
Source: PLoS Genet. 2014 Nov 6;10(11):e1004771. doi: 10.1371/journal.pgen.1004771 (PMC4222953; doi:10.1371/journal.pgen.1004771)
Supplement: Dataset S1 — RNA-seq data analysis pipeline for identifying significantly regulated A5C and A3C events. (ZIP) [file pgen.1004771.s018.zip › A5CA3C_pipeline/README.docx]

**A. Summary**

This pipeline extracts alternate 5’ and 3’ splice site choices (A5Cs and A3Cs) from RNA-seq datasets. For a single RNA-seq sample, all A5C and A3C events are simply enumerated as pairs of an upstream or a downstream alternate 5’ spice site (ss) joined with a single constitutive 3’ss (i.e. u5’ss-c3’ss and d5’ss-c3’ss pairs; A5Cs) and pairs of a single constitutive 5’ss joined with alternate upstream and downstream 3’ss (i.e. c5’ss-u3’ss and c5’ss-d3’ss pairs; A3Cs). When ≥2 RNA-seq samples are provided, the A5C and A3C lists are generated for each individual sample but each of these lists will contain inter-sample unions of the A5C and A3C events. Events differentially regulated between samples are then shortlisted using Fisher’s exact test.

**B. Dependencies and project directory structure**

You will need a Linux or an OS X Mac machine running Perl and R from a command line.

The pipeline is provided as a zip archive containing the file you are currently reading in both .docx and .txt formats, relevant Perl and R scripts, Annotated_UCSC_RefSeq_mRNA_exon_DB.bed file containing annotated library of known mouse exons, an empty Data_For_Fisher subdirectory (that will be used to hold data for Fisher testing as described in C.8-10 below), Exon_DB_Toolkit subdirectory with some optional Perl scripts (see E. below) and two BED junction files containing “toy” input data (located in the Toy_Input_Data subdirectory). The latter contains junctions from siControl- and siPtbp1-treated CAD samples mapped by TopHat to the mouse chromosome 19. This should be useful if you want to quickly test the pipeline before analysing your data. Simply unzip the archive into a project directory with a user-defined name and you are ready to go. All input and output data will be stored either in this directory or in corresponding subdirectories (e.g., Toy_Input_Data, Data_For_Fisher, or Exon_DB_Toolkit).

Note that the initial input of the pipeline consists of splice junction BED files generated by TopHat (or another aligner identifying splice junctions in RNA-seq datasets and outputting them as BED files). TopHat is not included in the bundle and will have to be provided separately as a local (http://ccb.jhu.edu/software/tophat/index.shtml) or Galaxy-based installation (https://usegalaxy.org/).

**C. Instructions**

**1.** Place junction BED files (any number of files is acceptable) into an appropriately named subdirectory inside the main project directory.

**2.** Open a terminal window and change PWD to the project directory.

**3.** Run bed_shifter.pl in the terminal window (requires Perl):

$ perl bed_shifter.pl <subdirectory containing input junction BED files>

This will convert original junction BED files into intron-centered BED files, the format required for the subsequent analyses. The output intron-centered BED files named exactly as the original BEDs will be saved in a new, script-generated subdirectory (IntCent-...) inside the main project directory. If needed, this subdirectory or/and the output files can be renamed by the user. For more information, run bed_shifter.pl without arguments.

**4.** Run unique_alternative5.pl to generate a non-redundant list of u5_d5_c3 junction alternative pairs present in the entire dataset. This is a time-consuming process that may take a few hours to complete. The output will be saved in the main project directory as a tab-delimited file ...-ua5-output.txt. For more information, run unique_alternative5.pl without arguments.

**5.** Run unique_alternative3.pl to generate a non-redundant list of c5_u3_d3 junction alternative pairs present in the entire dataset. This is a time-consuming process that may take a few hours to complete. The output will be saved in the main project directory as a tab-delimited file ...-ua3-output.txt. For more information, run unique_alternative3.pl without arguments.

**6.** Since the u5_d5_c3 junction alternative list generated in (4) will typically have entries corresponding to both bona fide A5C and other alternative splicing events, the A5C events will have to be enriched by running a5c_enricher.pl. The input for this script consists of the ...-ua5-output.txt file from (4) and a BED file containing annotated library of all known exons. En example of such library containing mouse exons from UCSC, RefSeq and mRNA databases is provided as a part of the pipeline bundle (Annotated_UCSC_RefSeq_mRNA_exon_DB.bed). The output file called Enriched... will additionally contain annotation information from Annotated_UCSC_RefSeq_mRNA_exon_DB.bed and it will be saved in the main project directory. For more information, run a5c_enricher.pl without arguments.

**7.** Since the c5_u3_d3 junction alternative list generated in (5) will typically have entries corresponding to both bona fide A3C and other alternative splicing events, the A3C events will have to be enriched by running a3c_enricher.pl. The input for this script consists of the ...-ua3-output.txt file from (5) and a BED file containing annotated library of all known exons. En example of such library containing mouse exons from UCSC, RefSeq and mRNA databases is provided as a part of the pipeline bundle (Annotated_UCSC_RefSeq_mRNA_exon_DB.bed). The output file called Enriched... will additionaly contain annotation information from Annotated_UCSC_RefSeq_mRNA_exon_DB.bed and it will be saved in the main project directory. For more information, run a3c_enricher.pl without arguments.

**8.** Run report_alternative5.pl to generate a summary table for A5C events. In addition to the Enriched... file from (6) this script requires the intron-centered BED files from (3). Note that the output file called ...ra5-output.txt will be saved in the Data_For_Fisher subdirectory. For more information, run report_alternative5.pl without arguments.

**9.** Run report_alternative3.pl to generate a summary table for A3C events. In addition to the Enriched... file from (7) this script requires the intron-centered BED files from (3). Note that the output file called ...ra3-output.txt will be saved in the Data_For_Fisher subdirectory. For more information, run report_alternative3.pl without arguments.

**10.** To calculate statistical significance of A5C and A3C events, run the fisherRa.R script in the terminal window (requires R):

$ Rscript fisherRa.R

This will run pairwise Fisher’s exact tests for all files stored in the Data_For_Fisher subdirectory. The output is saved as corresponding Fisher-... files in the main project directory. Note that the final A5C Fisher-... file for Toy_Input_Data should contain this Hps1-specific event significantly regulated upon Ptbp1 knockdown:

uc008hnx.1_annot1:_ep_annot2:_Mus_musculus_pale_ear_(ep_wild_type_allele)_mRNA,_partial_cds._exon:_2_0_chr19_42832155_r.

**D. Optional steps: BED stitching**

An optional script provided in the main project directory is called bed_stitcher.pl. It can be used to create non-redundant unions of BED files (either the original junction BEDs or intron-centered BEDs outputted by bed_shifter.pl; the script will also accept any other types of data). This might be useful if you want to combine junctions identified using two different aligners or two distinct sets of parameters of a single aligner (e.g., TopHat). For more information, run bed_stitcher.pl without arguments.

**E. Optional steps: Generating Exon Database**

The Exon_DB_Toolkit subdirectory contains 3 additional Perl scripts that should help you assemble your own annotated BED database of known exons. This will be required e.g. if you want to analyse human data or update the mouse exon DB with new entries. This BED database can then be used in place of the provided mouse-specific exon database Annotated_UCSC_RefSeq_mRNA_exon_DB.bed.

Examples of exon BED files (UCSC, RefSeq and mRNA track-based exons from mouse chromosome 19) and corresponding annotation files (UCSC and RefSeq annotations for mouse chromosome 19) are included in the Exon_DB_Toolkit subdirectory. This should allow you to quickly test this optional part of the pipeline.

Here is how an exon database can be assembled from scratch.

**1.** Go to the UCSC Table Browser (https://genome.ucsc.edu/cgi-bin/hgTables) and download BED files containing known exons. For example, select the following options to get all exons from UCSC known genes.

clade: Mammal

genome: Mouse

assembly: July 2007(NCBI37/mm9)

group: Genes and Gene Predictions

track: UCSC Genes

table: knownGene

region: genome

output format: BED – browser extensible data

output file: UCSC_exons.bed

file type returned: plain text

Click ‘get output’ and on the next page select

Create one BED record per: Exons plus 0 bases at each end

Click ‘get output’ to save this as a file.

To download RefSeq exons, select the following.

clade: Mammal

genome: Mouse

assembly: July 2007(NCBI37/mm9)

group: Genes and Gene Predictions

tract: RefSeq Genes

table: refGene

region: genome

output format: BED – browser extensible data

output file: RefSeq_exons.bed

file type returned: plain text

and then on the next page

Create one BED record per: Exons plus 0 bases at each end

Click ‘get output’ to save this as a file.

For mRNA track-based exons, select the following.

clade: Mammal

genome: Mouse

assembly: July 2007(NCBI37/mm9)

group: mRNA and EST

tract: mouse mRNAs

table: all_mrna

region: genome

output format: BED – browser extensible data

output file: mRNA_exons.bed

file type returned: plain text

and then on the next page

Create one BED record per: Blocks plus 0 bases at each end

Click ‘get output’ to save this as a file.

Save all files in a subdirectory where you plan to assemble the database (e.g. the Exon_DB_Toolkit subdirectory containing the relevant scripts).

**2.** Since the BED files downloaded in (1) will typically contain a number ofexon entries with identical chromosomal coordinates, these redundancies should be eliminated by running cis_nr_finder.pl. The output file will have Cis-... prefix added to the name of the original BED file and will contain a non-redundant list of exons. For more information, run cis_nr_finder.pl without arguments.

**3.** If you plan to combine exons extracted from different tracts (e.g., UCSC, RefSeq and mRNA exons) into a single database, you should run trans_nr_finder.pl specifying corresponding “target” and “depleter” BEDs. The output will be named Trans-<depleter>-depleted-<target> and it will contain only those exons from the original target file that are not coordinate-redundant with the depleter. If you plan to assemble exon database from ≥3 individual exon BED files, it may be necessary to repeat this step a few times to ensure that all BED components are mutually non-redundant. For more information, run trans_nr_finder.pl without arguments.

**4.** To append the cis/trans-non-redundant BEDs generated in the steps (2-3) with gene symbols and gene description, first download corresponding annotation data from the UCSC Table Browser (https://genome.ucsc.edu/cgi-bin/hgTables).

For example, select the following to get UCSC known gene exon annotation.

clade: Mammal

genome: Mouse

assembly: July 2007(NCBI37/mm9)

group: Genes and Gene Predictions

track: UCSC Genes

table: knownGene

region: genome

output format: selected fields from primary and related tables

output file: USCS_exons_annot.txt

file type returned: plain text

On the next page, tick the ‘name’ field in the mm9.knownGene table and the ‘geneSymbol’ and ‘description’ fields in the linked mm9.kgXref table. Click ‘get output’ to save this as a file.

For RefSeq exons, select.

clade: Mammal

genome: Mouse

assembly: July 2007(NCBI37/mm9)

group: Genes and Gene Predictions

track: RefSeq genes

table: RefGene

region: genome

output format: selected fields from primary and related tables

output file: RefSeq_exons_annot.txt

file type returned: plain text

On the next page, tick the ‘name’ field in the mm9.refGene table and the ‘geneSymbol’ and ‘description’ fields in the linked mm9.kgXref table. Click ‘get output’ to save this as a file that should look something like this:

#mm9.refGene.name mm9.kgXref.geneSymbol mm9.kgXref.description

NM_028778 Nuak2 NUAK family SNF1-like kinase 2 isoform B

NM_001195025 Nuak2 NUAK family SNF1-like kinase 2 isoform A

NM_001290393 n/a n/a

NM_008922 Prim2, DNA primase large subunit,

NM_175370 Als2cr12, amyotrophic lateral sclerosis 2 chromosomal,

NM_001290390 n/a n/a

NM_175642 Bai3, brain-specific angiogenesis inhibitor 3,

etc...

Save the annotation files in the Exon_DB_Toolkit subdirectory.

**5.** Run add_annot.pl script for corresponding BED and annotation file pairs. Note that running this script with an empty or wrong annotation file will add ‘n/a’ annotation placeholders. This may be useful to ensure consistent format of the exon database, for example when annotation is not readily available for some BED components of the future unified database file (e.g. mRNA-based exon BED file). The output of this script is called Annotated-... and it retains BED structure by concatenating corresponding annotation bits to the name of the exon through annot1: and annot2: spacers. For more information, run add_annot.pl without arguments.

**6.** The annotated cis- and trans-non-redundant component BEDs from (2-4) can then be manually assembled into a single exon database BED file with a user-specified name.
